# Cyanobacterial α-carboxysome carbonic anhydrase is allosterically regulated by the Rubisco substrate RuBP

**DOI:** 10.1126/sciadv.adk7283

**Published:** 2024-05-10

**Authors:** Sacha B. Pulsford, Megan A. Outram, Britta Förster, Timothy Rhodes, Simon J. Williams, Murray R. Badger, G. Dean Price, Colin J. Jackson, Benedict M. Long

**Affiliations:** ^1^ ARC Centre of Excellence in Synthetic Biology, Sydney, NSW, Australia.; ^2^ Research School of Chemistry, The Australian National University, Canberra, ACT 2601, Australia.; ^3^ Research School of Biology, The Australian National University, Canberra, ACT 2601, Australia.; ^4^ School of Environmental and Life Sciences, University of Newcastle, Callaghan, NSW 2308, Australia.

## Abstract

Cyanobacterial CO_2_ concentrating mechanisms (CCMs) sequester a globally consequential proportion of carbon into the biosphere. Proteinaceous microcompartments, called carboxysomes, play a critical role in CCM function, housing two enzymes to enhance CO_2_ fixation: carbonic anhydrase (CA) and Rubisco. Despite its importance, our current understanding of the carboxysomal CAs found in α-cyanobacteria, CsoSCA, remains limited, particularly regarding the regulation of its activity. Here, we present a structural and biochemical study of CsoSCA from the cyanobacterium *Cyanobium* sp. PCC7001. Our results show that the *Cyanobium* CsoSCA is allosterically activated by the Rubisco substrate ribulose-1,5-bisphosphate and forms a hexameric trimer of dimers. Comprehensive phylogenetic and mutational analyses are consistent with this regulation appearing exclusively in cyanobacterial α-carboxysome CAs. These findings clarify the biologically relevant oligomeric state of α-carboxysomal CAs and advance our understanding of the regulation of photosynthesis in this globally dominant lineage.

## Teaser

The carbonic anhydrase essential for carbon fixation in α-cyanobacteria is regulated by the Rubisco substrate RuBP.

## INTRODUCTION

A myriad of CO_2_ concentrating mechanisms (CCMs) have independently evolved to promote the rapid and efficient reduction of atmospheric CO_2_ into organic compounds. CCMs work to increase the local concentration of CO_2_ near ribulose-1,5-bisphosphate (RuBP) carboxylase/oxygenase (Rubisco), the primary carboxylase of the Calvin–Benson (CB) cycle, thereby increasing its substrate turnover and competitively inhibiting competing oxygenation reactions (*1*–*4*). These systems are an essential component of the global carbon cycle, catalyzing about half of global photosynthesis (*2*, *5*). The bacterial CCM, found in all cyanobacteria and some autotrophic bacteria, consists of two key elements: First, energy-coupled inorganic carbon (C_i_; primarily HCO_3_^−^ and CO_2_) transporters actively establish a concentrated pool of HCO_3_^−^ within a cytosol lacking free carbonic anhydrases (CAs); this HCO_3_^−^ then diffuses into proteinaceous microcompartments called carboxysomes that house CA and Rubisco (*2*, *3*). Here, the CA converts HCO_3_^−^ to CO_2_ to elevate luminal CO_2_, promoting Rubisco-catalyzed CO_2_ reduction (*6*). Bacterial CCMs have arisen in two distinct lineages: β-carboxysomes are found exclusively in β-cyanobacteria, containing form IB Rubisco with component genes encoded by the *ccm* operon and satellite loci (*7*), whereas α-carboxysomes are found in photoautotrophic α-cyanobacteria and several bacterial chemoautotrophs and are distinguished by the presence of form IA Rubisco and the clustering of carboxysome-associated genes into a discrete *cso* carboxysome operon (*8*).

Regulation of carbon fixation is essential for effective energy production. Indeed, the CCM is a notable example of how cells may induce physiological changes in response to environmental conditions. This adaptive capacity is a critical feature of these processes, involving regulation at the transcriptional and protein level, allowing the bacterial CCM to competitively support life in a range of ecological contexts (*9*, *10*). For example, CCM-related C_i_ transporters are regulated by gene expression and allosteric effectors (*11*–*13*), and carboxysome composition and morphology are responsive to environmental cues (*14*–*17*). Likewise, Rubisco content is transcriptionally regulated, and its activity is modulated by activases (*18*–*21*). However, little is known about how, or whether, the other enzymatic component of the carboxysome, the CA, is regulated (*22*).

The fundamental role of CAs in photosynthesis is well established (*6*). This versatile protein superfamily catalyzes the reversible hydration of CO_2_ [CO_2_ + H_2_O ⇌ HCO_3_^−^ + H^+^], comprising eight reportedly evolutionarily distinct classes (α, β, γ, δ, θ, η, ζ, and ι), distributed across the tree of life in a kingdom-nonspecific manner (*23*). In many cases, the enzyme directly supplies Rubisco with CO_2_, promoting its efficient reduction by ensuring reaction rate optimization through a constant, high concentration of the enzyme-substrate complex (*6*, *24*). Controlling Rubisco activity through buffering a bicarbonate pool in this way optimizes carbon fixation, coordinating CO_2_ assimilation rates with the generation of nicotinamide adenine dinucleotide phosphate/adenosine triphosphate produced in light reactions (*24*). Correspondingly, microbial and biochemical studies have established an absolute requirement for CA activity within the carboxysome (*25*, *26*) The α-carboxysome contains a highly divergent β-CA known as CsoSCA, characterization of which has occurred exclusively through the isoform from the chemoautotroph *Halothiobacillus neapolitanus* (*27*, *28*)*.* Indeed, structural and compositional studies have revealed sequence variation between cyanobacterial and proteobacterial carboxysome components and distinct carboxysome organization between these taxa (*29*–*34*). Given the differences in underlying metabolism between these photo- and chemoautotrophs, this has restricted our understanding of the CA-Rubisco feedback in α-cyanobacterial carboxysomes.

Here, we present a detailed biochemical, structural, and evolutionary analysis of a CsoSCA from a photoautotrophic cyanobacterium, *Cyanobium* sp. PCC7001 (*Cyanobium*), revealing previously unknown aspects of this isoform’s activity and molecular structure that form the basis for carboxysome regulation and organization. We found that, unlike the CA from the chemoautotrophic bacterium *H. neapolitanus* (*Hn*CsoSCA), the *Cyanobium* isoform (*Cy*CsoSCA) is regulated by the Rubisco substrate RuBP for activity, constituting a feedback loop at a key junction in α-cyanobacterial carbon metabolism. Detailed evolutionary analysis extends this, revealing that the sequence motifs for this regulation are not found in chemoautotrophic bacteria and expanding our understanding of α-cyanobacterial photosynthesis and CCM diversification more broadly.

## RESULTS

### *Cyanobium* CsoSCA requires RuBP for activity

**Fig. 1.**
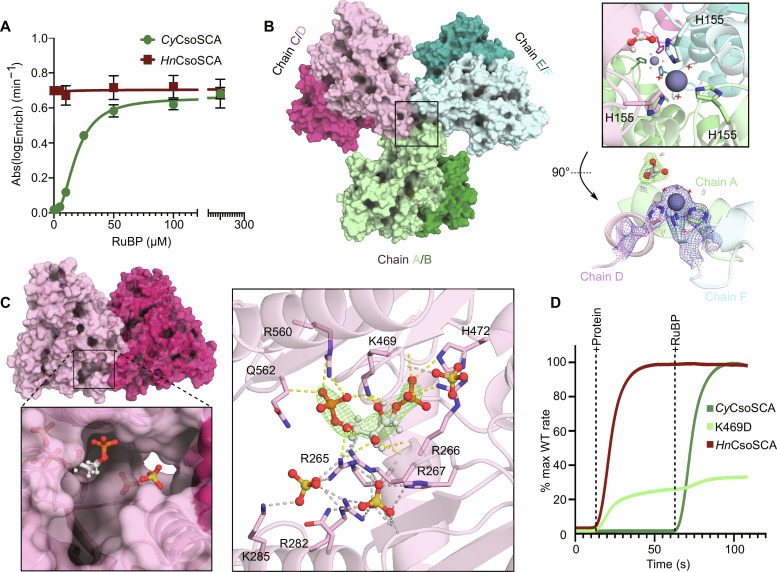
RuBP allosterically regulates *Cy*CsoSCA. (**A**) *Cy*CsoSCA and *Hn*CsoSCA activity as a function of RuBP concentration, measured by membrane inlet mass spectrometry (MIMS). Measurements reported are an average of three technical replicates, and error bars represent standard error. Curves were fitted using GraphPad Prism. (**B**) The homohexameric *Cy*CsoSCA structure solved to 2.3 Å (PDB ID: 8THM). The “trimer of dimers” arrangement is shown with dimers colored pink, green, or blue; monomers are indicated with different shades. The black square denotes the apical structural zinc atoms within the globular NTD with further detail shown in the right inset. Waters in the octahedral coordination sphere are shown as red crosses. Zinc ions are shown as gray spheres, and the bicarbonate ion is shown in ball-and-stick representation. Bottom: 2m*F*_o_ − d*F*_c_ density at key interacting residues is shown (1 s). Polder omit map (green) is shown at a contour level of 5.0 σ to highlight the bicarbonate ion density at the A/D/F apex. (**C**) The C/D dimer with a box highlighting the RuBP binding pocket of chain D. Right: The RuBP binding site is shown with the Polder omit map density (*75*) of RuBP overlayed at a contour level of 7.0 σ. Sulfate ions and RuBP are shown in ball-and-stick representation. Polar interactions are shown as dashed lines, RuBP interactions are shown in yellow, and other secondary/SO_4_ interactions are shown in gray. (**D**) The relative rate of *Cy*CsoSCA, the K469D mutant of *Cy*CsoSCA, and *Hn*CsoSCA are shown over time as a proportion of the maximum recorded reaction rate for each respective wild-type enzyme. Time points at which the protein and 100 μM RuBP were added to the MIMS cuvette are indicated. Curve is the mean of three technical replicates with the shaded area indicative of standard (not visible; see table S4). All structural and biochemical data were collected with the truncated *Cy*CsoSCA (fig. S1).

Despite homology to the constitutively active *Hn*CsoSCA (*27*), in our hands, *Cy*CsoSCA did not show detectable HCO_3_^−^ dehydration/CO_2_ hydration activity under standard assay conditions (*14*). This unexpected result indicated a potential additional requirement for *Cy*CsoSCA function. Given the previous observation of *Cyanobium* carboxysome function in vitro (*14*) and the established reliance on CA activity (*6*, *25*, *35*), we assessed *Cy*CsoSCA function under Rubisco assay conditions, where RuBP and Mg^2+^ are the key additional components. The addition of RuBP resulted in the concentration-dependent activation of *Cy*CsoSCA, with a *K*_M_ (Michaelis constant) for RuBP of 18 ± 2 μM, in a similar range to the *Cyanobium* Rubisco *K*_M_ for RuBP (36 μM) (*35*). Comparatively, *Hn*CsoSCA activity was unaffected by RuBP. Above 100 μM RuBP, *Cy*CsoSCA activity rates match those recorded for *Hn*CsoSCA (Fig. 1A). Notably, the *Cy*CsoSCA RuBP response curve is best described by a sigmoidal Hill function (*R*^2^ = 0.988), typically indicative of an allosteric activation mechanism. To mitigate precipitation, the disordered N-terminal region was removed from these isoforms (fig. S1). Subsequent analyses were conducted with these truncated forms, unless stated.

### *Cyanobium* CsoSCA structure reveals RuBP binding site and oligomeric state

To understand the structural basis for RuBP activation, CsoSCA was co-crystallized with RuBP. Diffracting crystals were obtained almost exclusively in saturating levels of RuBP with the final CsoSCA crystal structure solved through molecular replacement to a resolution of 2.3 Å (table S1). The resulting structure showed a homohexameric trimer of dimers consistently arranged in the asymmetric unit with *P*2_1_2_1_2_1_ symmetry (Fig. 1B). Size exclusion chromatography (SEC) corroborates that both *Cy*CsoSCA and *Hn*CsoSCA are primarily hexameric in solution (fig. S13), contrasting with previous observations (*27*). While the *Cy*CsoSCA dimer interface is highly reminiscent of *Hn*CsoSCA (*27*), additional contacts at the N-terminal domain (NTD) of each monomer mediate further quaternary assembly, forming two apices of the final hexamer (Fig. 1B and fig. S1). For comparison, the average buried surface area for individual *Cy*CsoSCA and *Hn*CsoSCA dimers is 1497.4 and 1458.4 Å^2^, respectively. An additional 460.7 Å^2^ of buried surface area at the trimeric apices is seen in the *Cy*CsoSCA complex solved here, with a total of 26,330 Å^2^ for the entire complex compared. A metal ion is evident at each apex, coordinated by a His_3_(H_2_O)_3_ octahedral coordination sphere, comprising His^155^ donated by a distinct monomer (Fig. 1B). This residue sits within a helical bundle in the NTD denoted here as the “hook motif.” The electron density and coordination geometry are consistent with a zinc ion. Density corresponding to a HCO_3_^−^ ion was observed at one of the trimer apices. While the pH of crystallization conditions favors bicarbonate, this species was not present at saturating levels under the crystallization conditions, which could explain its absence at the opposing apex.

Density consistent with RuBP was observed in all monomers within a positively charged pocket near the dimer interface that extends into the protein core (Fig. 1C, figs. S3 and S11, and tables S2 and S3). While variations in omit map density at these positions were observed, given the consistency of this density across each chain, the dependence on RuBP for crystallization, and the observation that RuBP was an allosteric activator of CA (Fig. 1), we were confident in the modeling of RuBP at this site. Two sulfate ions, likely from the crystallization solvent, could be modeled with high confidence at the entrance of this site in all monomers. While slight variations in RuBP ligand conformation are evident in each chain, contacts at Arg^266^, Lys^469^, and Arg^560^ are consistently observed (figs. S3 and S4). Most notably, RuBP curls around Lys^469^, mediating multiple H-bonds with the ligand. To confirm that this region is responsible for RuBP binding, we mutated Lys^469^ to Asp, the amino acid at the corresponding position in the constitutively active *Hn*CsoSCA isoform. This results in a biphasic activity profile with detectable CA activity evident in the absence of RuBP and a minor increase in activity upon addition of the ligand (Fig. 1D). This directly implicates K469 in the RuBP-mediated activation mechanism and further supports this region as the RuBP binding pocket.

### RuBP regulation of *Cyanobium* CsoSCA is allosteric

**Fig. 2.**
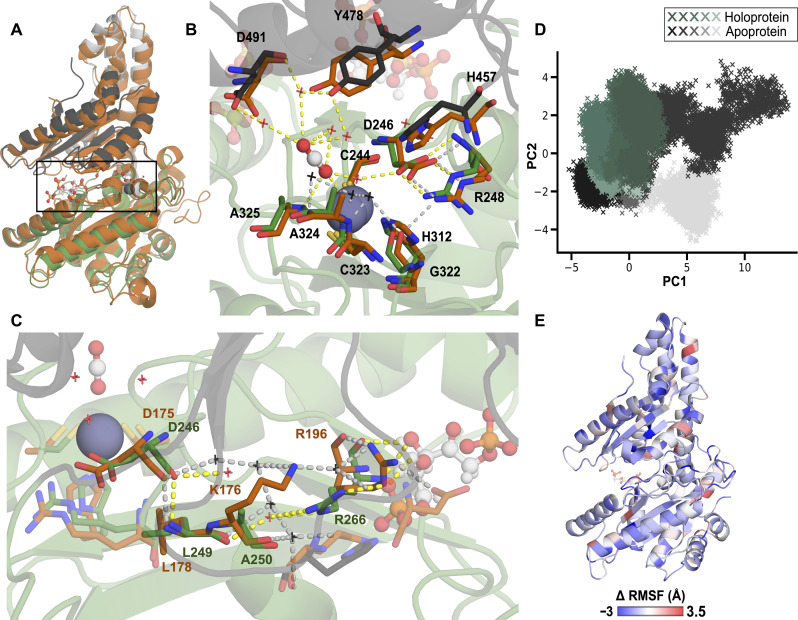
Active-site differences from *Hn*CsoSCA are key to allosteric activation of *Cy*CsoSCA. (**A**) Structural alignment of *Cy*CsoSCA and *Hn*CsoSCA monomers excluding the disordered N-terminal tails (Cα RMSD 1.5 Å). The *Cy*CsoSCA variant is colored by domain (white: NTD, green: catalytic domain, and dark gray: CTD). *Hn*CsoSCA (PDB ID: 2FGY) is shown in orange. The box denotes the active site and RuBP binding site region. (**B**) A close-up view of the *Cy*CsoSCA active site is shown with key residues in stick representation, colored as in (A). Corresponding *Hn*CsoSCA residues (orange) are overlaid. All ligands (CO_2_, RuBP, and SO_4_) are shown in ball-and-stick representation. Catalytically relevant water molecules are shown as crosses (waters in black are those in the *Hn*CsoSCA structure, and red indicates those in *Cy*CsoSCA). Dashed lines indicate polar bonds (gray indicates those between HnCsoSCA molecules and yellow denotes *Cy*CsoSCA interactions). Residue names are annotated according to the *Cy*CsoSCA structure. (**C**) The proposed allosteric network in *Cy*CsoSCA (top, green) and the corresponding region in *Hn*CsoSCA aligned. Key residues for each structure are annotated in orange (*Hn*CsoSCA) or green (*Cy*CsoSCA). (**D**) PCA comparing Cartesian coordinates of the *Cy*CsoSCA backbone in each MD simulation. Replicate simulations of *Cy*CsoSCA with and without RuBP are shown in green (holoprotein) and gray (apoprotein), respectively. (**E**) The average difference in RMSF values of holoprotein and apoprotein simulations (Δ RMSF), where a negative value indicates a greater RMSF (and thus more mobile residue) in the holoprotein. Values are mapped onto the *Cy*CsoSCA monomer. As the *Cy*CsoSCA structure solved here was used for MD simulations, it excludes the 116-residue disordered N-terminal tail (fig. S1).

Given the sigmoidal *Cy*CsoSCA activation curve and RuBP binding site distinct from the active site (Fig. 1), we hypothesized that RuBP acts as an allosteric activator. The β-CA family is the only CA family known to exhibit allostery to date (*36*). Alignments between *Cy*CsoSCA and a structure of a previously characterized type II β-CA bound to the allosteric bicarbonate ion show that RuBP engages distinct residues and sits further from the active site, indicating a distinct regulatory mechanism (fig. S6). The RuBP site overlays with the region of the CsoSCA C-terminal domain (CTD) that has lost the second symmetric catalytic zinc site seen in canonical β-CAs, likely following gene duplication and divergence of the catalytic domain (*27*). A structural analysis of the *Cy*CsoSCAs and *Hn*CsoSCAs was conducted to identify potential allosteric networks within the cyanobacterial variant. *Cy*CsoSCA monomers align well with the canonical *Hn*CsoSCA [37.2% sequence identity and Cα root mean square deviation (RMSD) of 1.5 Å; Fig. 2A], and the three domains (NTD, catalytic domain, and CTD) (*27*) are evident (Fig. 2A). The Cys_2_His(H_2_O) tetrahedral coordination of the catalytic zinc ion typical of β-CAs is maintained, and the overarching active site is highly homologous between each CsoSCA isoform. The Asp-Arg dyad between active-site residues Asp^246^ and Arg^248^ precludes the inactive Cys_2_HisAsp coordination sphere across all *Cy*CsoSCA monomers, reinforcing the classification of CsoSCA as type I (*27*). This type of β-CA has not previously been associated with allostery. Manual inspection of H-bonds within the protein identified a network linking the catalytic Asp^246^ backbone and the Arg^266^ side chain that, in turn, binds RuBP, mediated by a water molecule and Leu^249^ backbone groups (Fig. 2B). In the corresponding region in the constitutively active *Hn*CsoSCA, Lys^179^ (Ala^250^ in *Cy*CsoSCA) occupies this space, coordinating a more extensive interaction network reinforced by multiple water molecules. Notably, Arg^196^ (Arg^266^ in *Cy*CsoSCA) binds Asp^409^ (Lys^469^ in *Cy*CsoSCA) identified above as a key determinant in RuBP-dependent activity (Fig. 2C).

Molecular dynamics (MD) simulations of the *Cy*CsoSCA structure (300-ns replicates) were conducted in the presence (holoprotein) and absence (apoprotein) of RuBP to assess for conformational changes upon ligand binding to evaluate the allosteric effect of RuBP. Analyses of replicate simulations are consistent with *Cy*CsoSCA accessing different conformational landscapes when RuBP is present or absent. Principal components analysis (PCA) of the trajectories highlights differences between conformations sampled in apo- and holoprotein states, with holoprotein replicates converging on a distinct cluster as the simulations equilibrate (Fig. 2D and fig. S8). On average, residues in the apoprotein trajectories across the structure had root mean square fluctuations (RMSFs), indicating that they are more mobile (Fig. 2E). While it is difficult to ascribe a molecular mechanism with confidence, these results support a model in which RuBP stabilizes *Cy*CsoSCA by establishing an internal H-bond network, promoting access to the active conformation.

### Sequence patterns indicate that allosteric CsoSCA is limited to cyanobacteria

**Fig. 3.**
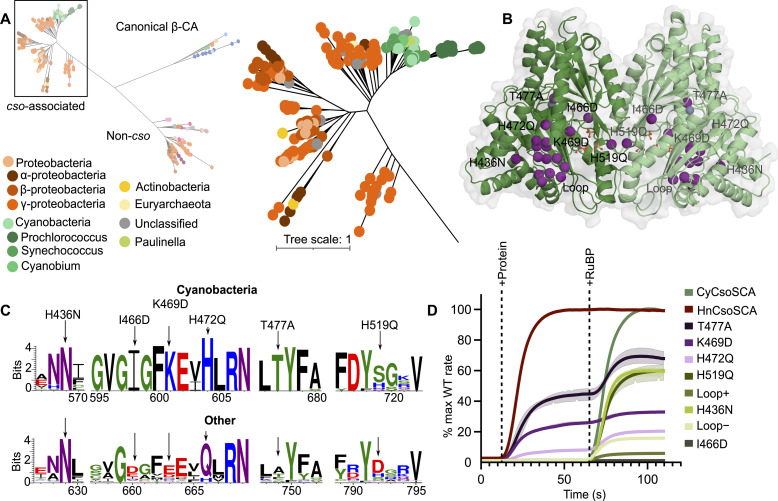
CsoSCA sequence analysis and mutagenesis. (**A**) Unrooted ML phylogeny of 504 CsoSCA sequences produced by IQ-Tree including 19 canonical β-CAs as an outgroup (annotated as β-CA). The cluster containing members associated with the *cso* operon is shown in detail. Tips are colored by taxonomy according to the legend. Tree scale refers to the number of substitutions per site. See the Supplementary Materials for complete tree annotation and documentation. Complete tree files and associated alignments provided as Supplementary data files. (**B**) Sites targeted for mutagenesis shown as purple spheres on the *Cy*CsoSCA dimer. (**C**) Sequence logos based on α-cyanobacterial *cso*-associated CsoSCA sequences (Cyanobacteria) or other bacterial *cso*-associated CsoSCA sequences (Other) of key sites targeted for mutation. Residues colored by chemistry, and logos were generated using WebLogo3. Mutations are as represented above the logo. (**D**) Activity assays of targeted mutants relative to the maximum rate recorded for wild-type *Cy*CsoSCA. *Hn*CsoSCA activity is also shown for comparison as a proportion of its maximum recorded rate. HCO_3_^−^ dehydration activity was recorded using MIMS after the addition of CsoSCA variants (+Protein) and upon addition of 100 μM RuBP (+RuBP). Three technical replicates were recorded for each variant, and the standard deviation is indicated by shading (error on some samples not visible due to scale; see table S4). All mutants created in the truncated *Cy*CsoSCA wild-type background lacking the 116 residue N-terminal disordered region (fig. S1).

We sought to investigate the prevalence of RuBP allostery within the broader CsoSCA protein family by mapping the sequence diversity of the family to *Cy*CsoSCA functional variation. To examine CsoSCA divergence, maximum likelihood (ML) phylogeny was inferred from a curated sequence database of the CsoSCA Pfam (PF08936) (Fig. 3A and fig. S10). Cyanobacteria form a clear, tight cluster distinct from other bacterial species, supported by a high bootstrap value, as seen in related studies (*30*, *37*, *38*). We hypothesized that RuBP regulation may be specific to photoautotrophs, evolving as the *cso* operon adapted to these organisms’ light-dependent metabolic requirements relative to ancestral chemoautotrophic α-carboxysomes (*38*).

To test this, concurrent approaches based on rational design and directed evolution were used to discern key residues involved in RuBP regulation. The conservation of these positions was then assessed across the CsoSCA protein family (Fig. 3). Candidate residues for targeted mutagenesis were chosen through successive steps of analyzing the sequence and structure of *Cy*CsoSCA and *Hn*CsoSCA to locate residues with distinct biophysical properties near the RuBP pocket. Final mutations were made at sites that differed between the two characterized isoforms and had varying levels of conserved difference between cyanobacterial taxa and other bacterial CsoSCA isoforms more broadly (K469D, H472Q, I466D, and H436N). Manual sequence inspection revealed a loop region in *Hn*CsoSCA (position in *Hn*CsoSCA) that contained an insertion conserved across cyanobacterial species (position in *Cy*CsoSCA) but absent or nonconserved in other proteins. Mutants were created in the *Cy*CsoSCA background with either a deletion of this loop (loop deletion) or with the corresponding *Hn*CsoSCA loop sequence substituted at this site (loop insertion). Alongside this approach, *Cy*CsoSCA was also randomly mutagenized, facilitating a broader exploration of the sequence space involved in this activation mechanism.

Mutants were screened using an in-house CA knockout *Escherichia coli* strain (*39*) for variants with CA activity independent of RuBP.Activity assays of these mutants revealed that, in addition to K469D, an H472Q mutation (targeted approach) and T477A (random approach) also resulted in a biphasic activity profile with CA function independent of RuBP (Fig. 3D). Other mutations resulted in either reduced or undetectable CA activity, making their effects on RuBP dependence specifically difficult to infer. Sequence-based analyses show that residues with apparent involvement in RuBP dependence are well conserved in α-cyanobacteria but absent or nonconserved in other taxa (Fig. 3C). The conservation of sites underpinning RuBP dependence is consistent with this regulation existing primarily, if not exclusively, in photoautotrophic CsoSCA variants.

### The unique N-terminal oligomerization domain is exclusive to carboxysomal CAs

**Fig. 4.**
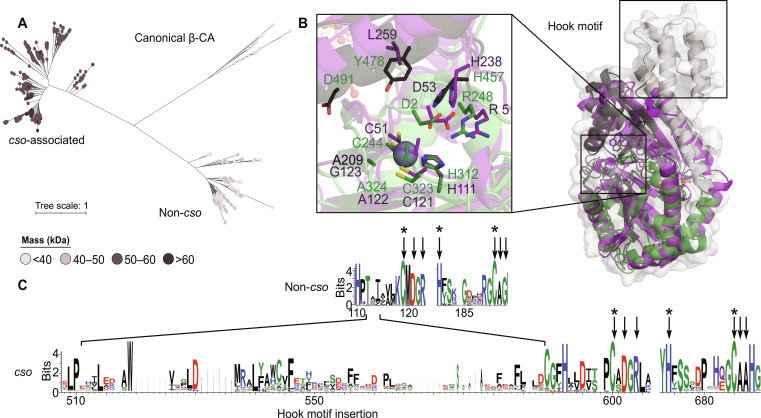
The NTD is unique to carboxysome operon–associated CsoSCA homologs. (**A**) ML tree of the CsoSCA Pfam (PF008936) and canonical β-CA with nodes colored by predicted mass as per the legend. (**B**) Structural alignment of Cα backbones of *Cy*CsoSCA (NTD: gray, catalytic domain: green, and CTD: black) to an AF2-generated model of a candidate non-*cso* sequence (UniProt ID: A0A080M7C6; purple) with a close-up view of key active-site residues. The insertion exclusive to *cso*-associated sequences that encodes the NTD hook motif is annotated. (**C**) Sequence logo of alignments of either CsoSCA members associated with *cso* operons (*cso*) or non-*cso* sequences. The *cso* insertion encoding the NTD hook motif is annotated. Key catalytic residues are indicated with arrows, and zinc-binding residues are indicated with asterisks.

Further bioinformatics analysis of the CsoSCA protein family revealed an orphan cluster within this β-CA clade (Fig. 4). Manual inspection of the gene neighborhoods of these sequences demonstrates that they are not associated with the *cso* operon, appearing instead within other metabolic gene clusters, often associated with nicotinamide adenine dinucleotide or [Fe-Ni] hydrogenases or permeases (fig. S15). Structural modeling of “non-*cso*” sequences and subsequent structure-based searches using Foldseek (*40*) and DALI (*41*) shows that these non-*cso* sequences align preferentially to the published *Hn*CsoSCA structure with high confidence relative to other β-CAs (table S5). These sequences appear to have lost the typical β-CA twofold symmetry, containing only one predicted zinc-binding site per pseudo-dimer, a defining feature of canonical CsoSCA sequences (Fig. 4). However, all non-*cso* sequences are notably shorter than carboxysome-associated variants. This is underlined by a consistent insertion in *cso-*associated sequences within the NTD, which encodes a hook-like bundle of α helices dubbed the “hook” motif (Fig. 4) shown here to facilitate structural zinc binding and oligomerization (Fig. 3). This is consistent with NTD presence and, thus, hexamer formation being a more recent adaptation unique to carboxysome-encapsulated variants of this family.

### Carboxysome functional modeling indicates an adaptive advantage for RuBP-regulated CsoSCA

**Fig. 5.**
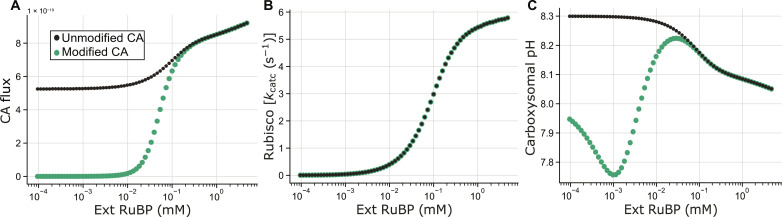
Results of a reaction-diffusion model adapted to emulate *Cyanobium* α-carboxysome function with an RuBP-dependent CA (modified CA; green dots) or a constituently active CA (unmodified CA; black dots). (**A**) In the modified model, carboxysomal CA activity was altered based on data shown in Fig. 1A to be dependent on carboxysomal RuBP concentrations [Ext RuBP (mM)]. “CA flux” is indicative of CA activity, confirming RuBP dependence in the modified model. (**B**) Rubisco carboxylation turnover rates {Rubisco [*k*_catc_ (s^−1^)]} as a function of modeled cellular RuBP concentrations [Ext RuBP (mM)] in the modified and unmodified systems. (**C**) Modeled carboxysomal pH is plotted as a function of the modeled cellular RuBP content [Ext RuBP (mM)] and indicates a decrease in carboxysomal pH when CA function is allosterically controlled by RuBP, with potential to aid Rubisco function as described previously (*35*).

The results presented above support the hypothesis that CsoSCA RuBP dependence is a fixed trait unique to cyanobacterial α-carboxysome systems. To determine whether this feature emerged as an adaptive or neutral change in CsoSCA variants, we aimed to discern a functional benefit for RuBP regulation within the α-cyanobacterial system. An in vivo assessment of CsoSCA regulation is currently intractable, with no effective genetic transformation techniques reported to date. Instead, we modified our carboxysome steady-state diffusion model (*35*), incorporating kinetic data presented in Fig. 1A to compare the activity of a *Cyanobium* carboxysome with and without an RuBP-dependent CA (Fig. 5A). While second to physiological data, this approach permitted insights into any direct effects this regulation may have on core enzyme activity and metabolite flux of the *Cyanobium* α-carboxysome. No substantial changes in Rubisco carboxylation or oxygenation rates were observed between the standard model and one incorporating an RuBP-dependent CA (Fig. 5B and fig. S12). We note that modeling of the unmodified *Cyanobium* carboxysome here had a slightly alkaline carboxysome pH compared with the observation of an acidic carboxysome in our previous modeling (*35*). This is due to the use of *Cyanobium* Rubisco kinetics, the concentration of HCO_3_^−^ tested here (20 mM), and a modification of the Rubisco active-site concentrations compared with previous modeling [a reduction from 10 to 5.7 mM to fit recent estimates (*29*)]. Applying RuBP dependence on CA activity in this model causes a shift to a more acidic carboxysomal lumen under low cellular RuBP levels. This implicates a regulated CA in maintaining an acidic carboxysome lumen under low-RuBP conditions in the *Cyanobium* system, likely experienced as light levels fluctuate throughout the diurnal cycle (*14*). Though the current model framework does not permit direct modeling of the effect of pH on carboxylation over time, previous work has shown an optimum for Rubisco activity (*42*) and a pH effect on net CO_2_ supply within carboxysomes (*35*, *43*). Together, and in lieu of direct experimental data, the effect of RuBP-mediated CA modulation may contribute to CCM function by maintaining optimal pH conditions within the carboxysome for efficient CO_2_ fixation.

## DISCUSSION

The α-carboxysomal CCM enables efficient C_i_ fixation across a diverse range of microorganisms, comprising a major component of the global biosphere (*5*, *44*). Given the essential nature of CAs in this system (*6*), characterizing the CsoSCA variant present in photosynthetic organisms and understanding its evolutionary trajectory provide important insights into the emergence and function of bacterial CCMs. Data presented here are consistent with RuBP allosterically activating *Cy*CsoSCA and the likely confinement of this property to α-cyanobacteria. A hexameric trimer of dimers quaternary state is also described, coordinated by NTD contacts with structural zinc ions that appear only in carboxysome-associated members of the CsoSCA protein family.

### A distinct paradigm for CA allosteric regulation

Allosteric regulation of CAs is rare, particularly allosteric activation. The β-CA family is the only CA family known to exhibit allostery, with HCO_3_^−^ acting as both a substrate and an inhibitor in type II members. Binding of the HCO_3_^−^ disrupts the “gatekeeper” Asp-Arg dyad, leading to the Asp-Zn bond that displaces the catalytic H_2_O, leading to inhibition (*36*, *45*–*47*). In addition to activating the enzyme, the RuBP binding pocket presented here appears distinct from these previously characterized sites, engaging different residues and sitting further from relative active sites (figs. S3 and S6). Indeed, the RuBP site sits near the defunct active site of the CTD within the CsoSCA pseudo-dimer, previously identified as a highly divergent catalytic domain that has lost key zinc-binding sites and catalytic loops (*27*). The duplication and divergence of domains, particularly at protein termini, are a common motif in protein evolution (*48*). While seemingly acting as a regulatory domain in the cyanobacterial CsoSCA, it is unclear whether the CTD takes on alternative, more cryptic regulatory roles in RuBP-independent CsoSCA isoforms or indeed in the smaller uncharacterized non-*cso* isoforms.

We propose an allosteric activation mechanism in which RuBP binding establishes an internal H-bond network near the core of *Cy*CsoSCA that has a broadly stabilizing effect on the protein; this, in turn, promotes access to the active conformation. RuBP binding engages Arg^266^ in H-bonding, establishing a H-bonding network that links to active-site loops (Fig. 3). The equivalent network in *Hn*CsoSCA is more extensive involving more residues, specifically negatively charged residues that are absent in the *Cy*CsoSCA isoform. Specifically, in *Hn*CsoSCA, the Arg^266^ equivalent (Arg^196^) is stabilized by analogous interactions with Asp^409^ (Lys^469^ in *Cy*CsoSCA), thereby negating the ligand-binding requirement for activation. This proposed mechanism is also consistent with the observed biphasic activity profile of mutants, many of which introduce a relatively negative charge to this site, most notably K469D (Fig. 3). However, it remains difficult to rationalize the relatively large increase in RuBP-independent activity observed in the *Cy*CsoSCA^T477A^ mutant. While Thr residues are typically known to stabilize β sheets, given this site is buried, it may be that this exchange reduces secondary structure strain and enhances protein core packing while having more minimal disruption to the overarching protein fold than other mutants (*49*). Consequently, T477A would result in increased apoprotein stability and a wild-type-like RuBP pocket, leading to high levels of ligand-independent activity and a notable increase with RuBP. Although the involvement of the Asp-Arg dyad destabilized by HCO_3_^−^ does seem reasonable in the context of *Cy*CsoSCA given its established role as an allosteric switch (*36*, *45*), we could not establish this as the clear mechanism for allosteric signal propagation. Notably, carboxysomal CAs in both α- and β-carboxysomes exhibit various levels of redox regulation, all appearing to be inactivated by reducing conditions, typical of the cytoplasm, and activated upon oxidation, as expected to occur within the distinct carboxysomal lumen environment (*22*, *28*, *50*). Though a detailed redox characterization of CsoSCA is lacking in the literature; this appears to be distinct from RuBP activation (fig. S5). Future efforts to elucidate this redox regulation will be required for a complete understanding of carboxysomal CAs.

### An adaptive advantage for an RuBP-regulated CA in some photoautotrophs

The conservation of residues central to RuBP-dependent activity corresponds with the divergence of the α-cyanobacterial clade in the CsoSCA phylogeny (Fig. 3). This suggests that allosteric RuBP activation is either a neutral or adaptive change uniquely fixed in α-cyanobacteria relative to other α-carboxysomal taxa. RuBP allostery appears infrequently in the literature, primarily characterized in Rubisco adjacent proteins such as the AAA+ red-type activase Cbbx (*51*). In these cases, it is proposed to synchronize the activity of such proteins with Rubisco, hinting at an intricate regulatory cycle to ensure efficient Rubisco function. Indeed, modeling and experimental data have established a similar functional link between CA and Rubisco, demonstrating that Rubisco function in carboxysomes is intrinsically dependent on CA activity (*6*, *25*, *35*). This type of posttranslational regulation would enable more rapid responses to transient metabolic signals, directly synchronizing CB cycle fluxes with Rubisco-mediated carbon fixation.

We propose that RuBP-dependent allostery may be linked to large fluctuation in cellular RuBP observed in photosynthetic α-cyanobacteria but absent in proteobacterial systems such as *H. neapolitanus* that are likely under more constant environmental substrate supply (*14*, *52*). Using a carboxysome reaction-diffusion model, we previously identified that carboxysomes may require molecular mechanisms that modulate internal pH as RuBP concentrations vary to the detriment of Rubisco function (*35*). Modification of this model to allow for allosteric activation of CA in *Cyanobium*-like carboxysomes indicates that such regulation ameliorates this effect, resulting in a modulation of carboxysome pH without any change in Rubisco carboxylation (Fig. 5 and fig. S12). Thus, the emergence of RuBP regulation in these systems may have been prompted by a requirement for more fine-tuned control over carboxysomal H^+^ concentrations in photoautotrophic systems that are subject to more drastic cellular RuBP fluctuations across the diurnal cycle. That the protein can be switched from a constitutive to an autoinhibitory isoform in one residue change highlights the evolutionary pliability of this protein, suggesting that the evolution of allostery from a constitutively active isoform may have arisen from a relatively small sequence level change in ancestral photoautotrophic hosts in the presence of new fitness pressures. This raises the question as to why β-carboxysomes, present exclusively in photosynthetic cyanobacteria, do not appear to have the same requirement (*50*, *53*). In the absence of a model, computational or otherwise, that can explicitly measure this, we speculate that the larger size of β-carboxysomes (~100 versus ~250 to 500 nm in diameter), and thereby smaller surface area:volume ratio, relative to α-carboxysomes may lead to diffusional differences between these structures (*35*, *38*). Specifically, the smaller α-carboxysomes likely experience greater diffusional rates and faster achievement of homeostasis. This may mean that β-carboxysomes do not need to respond rapidly to internal species fluctuations. An RuBP-regulated CA may offer a mechanism to enable rapid homeostasis in smaller compartments. Given this, we conclude that the emergence of an RuBP-dependent CA comprises a key molecular step in the adaptation of the α-carboxysome within cyanobacterial lineages.

### The α-carboxysomal CA is hexameric in solution

The oligomeric state of enzyme cargo is an important detail of bacterial microcompartment systems, providing insights into cargo organization and interaction networks within the shell. Here, we show that *Cy*CsoSCA and *Hn*CsoSCA are both hexameric in solution, not dimeric as previously described (*27*), and that this quaternary structure is mediated by the globular NTD (Fig. 1 and figs. S1 and S2). This hexamer likely eluded detection in the *Hn*CsoSCA structure solved previously due to an accidental mutation that introduced an artificial second zinc site within the globular NTD of each monomer (*27*). This would have inhibited the structural zinc interactions that we observe as essential for the coordination of dimers within the hexameric complex (Fig. 1 and fig. S13). It is interesting that the previous study did not report any difficulty in purifying this *Hn*CsoSCA mutant and express the complete disordered N-terminal without serious aggregation, in contrast to our and others’ accounts of wild-type isoforms (*30*) (fig. S1). It may be that hexamerization increases the propensity of CsoSCA to precipitate by enhancing the local density of disordered N-terminal tails that may self-interact, resulting in aggregates at high concentrations, or perhaps phase separation as has been reported between interaction partners in the system (*30*, *54*). Given the stability and dominance of hexamers in vitro (fig. S13), it is plausible that this conformation, or perhaps a mix of hexameric and dimeric forms, exists in native carboxysomes. Hopefully, the identification and characterization of the hexameric CsoSCA in this work will aid future efforts to elucidate the internal architecture of α-carboxysomes in vivo.

CsoSCA is now known to interact with form IA Rubisco through interactions between the CsoSCA N-terminal disordered tail in a manner reminiscent of the α-carboxysome structural protein CsoS2 (*30*, *50*). Though the conservation of this binding motif is limited, the N-terminal disordered tail likely only contains a single binding motif. Comparatively, CsoS2 contains multiple Rubisco binding motifs, thereby enhancing the valency and strength of the interaction. Considering this, a hexameric assembly would enhance the local concentration of Rubisco interaction motifs and, thus, multivalency of the system to promote CsoSCA-Rubisco binding. In this way, the emergence of the NTD may constitute a molecular marker for the early association of an ancestral CsoSCA-Rubisco complex, hypothesized as a likely carboxysome evolutionary precursor (*35*, *55*).

As we begin to resolve the evolutionary trajectories of bacterial CCMs, a detailed understanding of structural variation of core system components and how this relates to function will be essential for resolving plausible evolutionary routes. We have identified a distinct divergence between α-cyanobacteria and other α-carboxysome taxa, presenting a paradigm for CO_2_ fixation in photoautotrophic α-cyanobacteria that hinges on the regulation of CsoSCA by RuBP. These results highlight the intrinsic role of CAs in photosynthesis, comprising an evolutionarily pliable enzyme at the bottleneck of key reactions in the carbon metabolism of many diverse organisms. This aspect of carboxysome function and evolution must be considered in future biotechnological applications that seek to adapt such systems, particularly efforts to transplant the bacterial CCM into photoautotrophic crop species.

## MATERIALS AND METHODS

### CsoSCA expression constructs

In initial expression tests, we found that the N-terminal disordered region (fig. S1) causes severe aggregation, inhibiting expression and purification and thus was removed in all CsoSCA isoforms discussed in this investigation. This propensity to aggregate was also noted by previous work in their supplemental methods addendum (*30*). The wild-type *Hn*CsoSCA (O85042 with first 49 residues removed) and *Cy*CsoSCA [WP_071778263.1 with first 116 residues removed (fig. S1)] were codon-optimized for expression in *E. coli* and cloned into the pHue expression system (*56*). These N-terminal truncations removed the nonconserved disordered N-terminal region of these proteins. While these disordered regions are known to be essential for CsoSCA encapsulation during carboxysome biogenesis, they are assumed not to have a role in CA activity or enzyme function (*30*, *57*). Increased severity of aggregation in our work and in previous works relative to the original *Hn*CsoSCA study may be due to the inadvertent mutation in previous works that prohibits hexamer formation (fig. S11) (*27*, *30*). The presence of these larger quaternary complexes would increase the local concentration of disordered regions and perhaps lead to greater condensation/aggregation in solution (fig. S2). Codon-optimized genes encoding *Cy*CsoSCA mutants were synthesized through Twist Bioscience and cloned into the pHue vector using standard Gibson assembly methods (*58*). Primers used are listed in table S5.

### CsoSCA random mutagenesis

To create random CsoSCA mutants with RuBP-independent activity profiles, random base changes were introduced using error-prone polymerase chain reaction (epPCR), and the resulting libraries were screened for desired activity profiles. The GeneMorph II Kit (Agilent, catalog no. 200550), containing the Mutazyme II DNA polymerase blend, was used in the epPCR reaction to generate mutant libraries using conditions specified in the manual for medium rates of mutagenesis. Primers were designed to enable a one-pot cloning reaction of resulting PCR fragments into a pET16 expression vector (table S6). The resulting one-pot reaction mix was dialyzed using the MF-Millipore 0.25 μm MCE Membrane (Merck, Ireland) to remove salts and transformed into dense aliquots of electrocompetent DH5α *E. coli* cells. The library was plated and grown overnight at 22°C until pinprick colonies were visible. Plates were then washed with 5 ml of lysogeny broth (LB) to recover colonies and plasmids extracted using the QIAprep Spin Miniprep Kit (Qiagen, catalog no. 27104). The “DCAKO” cell line is an *E. coli* strain, created in Price lab, in which both native CA genes have been knocked out. Consequently, cells cannot grow under ambient conditions. Five microliters of this library was transformed into dense aliquots of electrocompetent DCAKO *E. coli* cells to screen the mutant library for members with the desired activity profile (activity independent of RuBP). Transformed cells were grown in triplicate plates at 37°C/ambient or 37°C/4% CO_2_ conditions. Colonies that appeared on plates grown under ambient conditions were re-grown in liquid cultures at 37°C/4% CO_2_ and plasmids extracted for sequencing. Resulting mutant *csoSCA* sequences were analyzed using the Geneious Prime software package. Mutants that permitted DCAKO growth under atmospheric conditions were sequenced, and a representative pool was chosen for crude activity assays to confirm CsoSCA activity independent of RuBP. Three residues (H519Q, T477A, and N278D) consistently corresponded to RuBP-independent CA activity, and all had notably different chemistry to the analogous sites in *Hn*CsoSCA. However, in the mutant library, these sites were always present within a small background of other site changes. To assess the effect of these residues directly, single-site mutants were generated using custom primers and cloned into the pHue expression system (table S6). The N278D mutant was incredibly difficult to clone and consistently precipitated during expression, leading us to abandon it for further analysis.

### Protein expression and purification

The USP2-pHue expression system was used for heterologous expression of CsoSCAs (*56*). NEB T7 Express pLysY chemically competent *E. coli* cells were transformed with a pHue plasmid containing the corresponding CsoSCA sequence. Single colony glycerol stocks (1:1 ratio of 40% glycerol and cell culture) were used to inoculate 3 ml of cultures in LB media supplemented with ampicillin (100 μg/ml; LB + Amp) and grown overnight at 37°C shaking at 200 rpm. From these overnight cultures, 1 ml was used to inoculate 25 ml of fresh LB + Amp, which, in turn, was incubated at 37°C for 6 hours in LB media, and 10 ml of this was then used to inoculate 500 ml of fresh LB + Amp. This was grown at 37°C for 2 hours, followed by induction with 100 μM isopropyl-β-d-thiogalactopyranoside and further incubation overnight at 28°C. Cell cultures were pelleted and stored at −80°C until required.

Cells were thawed, resuspended in binding buffer [50 mM tris (pH 7.8), 300 mM NaCl, and 25 mM imidazole], incubated at room temperature, and shaken with 10% rLysozyme (EMD Millipore Corp, USA) and 0.2 μl of Turbonuclease (Sigma-Aldrich) for 30 min. Cells were lysed with three passes of the Emulsiflex (Avestin, USA). Lysate was clarified by centrifugation (16,000*g* for 30 min at 4°C), and the soluble fraction passed through a 0.44-μm syringe filter before application to a pre-equilibrated 5-ml HisTrap HP column (GE Healthcare). The column was washed with 50 ml of binding buffer and the target protein was eluted in elution buffer [50 mM tris (pH 7.8), 300 mM NaCl, and 500 mM imidazole]. Fractions containing protein were pooled and concentrated to a maximum of 5 ml using a centrifuge filter (Amico Ultra-15 Centrifugal Filter Unit) and buffer-exchanged using a PD-10 column (GE Healthcare, lot 9760001) into size exclusion buffer [SEC buffer; 50 mM tris (pH 7.8) and 300 mM NaCl]. Samples were incubated with prepurified USP2 at a 1:10 protein-of-interest:USP2 molar ratio and incubated with rocking at 4°C for at least 12 hours with 2 μl of β-mercaptoethanol. Samples were then passed over clean HisTrap columns equilibrated with 50 ml of SEC buffer to remove protease. Flow through was collated and concentrated through centrifugation as above and further purified by SEC on a HiLoad 26/600 Superdex 200 Column (GE Healthcare), eluting in SEC buffer. Protein purity was confirmed by SDS–polyacrylamide gel electrophoresis, and protein concentrations were determined spectrophotometrically by measuring *A*_280_ (absorbance at 280 nm) using a NanoDrop One (Thermo Fisher Scientific) and molar absorption coefficients generated by ProtParam (http://expasy.org/tools/protparam.html) (*59*).

### CA activity assay

All CA activity was measured using the MIMS technique and relevant equations were adapted for pure protein samples (*26*). This is based on measuring the loss of ^18^O_2_ from a labeled C_i_ source to water through the CA-catalyzed hydration and dehydration of CO_2_ and HCO_3_^−^, respectively. Briefly, NaH^13^CO_3_ is incubated for 24 hours with H_2_^18^O, creating a ^13^C-labeled carbon source enriched with ^18^O. The change in the ^18^O enrichment of CO_2_ species is measured after chemical equilibrium was reached before and after the addition of enzyme. The ^18^O atom fraction (the atom % enrichment) was calculated at each point by summing the related carbon species determined by mass spectrometry. CA activity is reported as the rate of decline of the atom % enrichment, calculated from the slope of the change in log(atom % enrichment) over time (Log_Enrich_ min^−1^). This gives a measure of the first-order rate constant. As this refers to the decline of ^18^O, this rate becomes increasingly negative with CA activity. For simplicity, we display the absolute value here. All assays were conducted with 0.1 μM protein and 2.7 mM heavy isotope bicarbonate (NaH^13^C^18^O_3_) in CA buffer [50 mM EPPS-NaOH and 20 mM MgCl_2_ (pH 7.8)] in a final cuvette volume of 600 μl. CsoSCA has previously been shown to be inactivated by reducing agents, indicative of an oxidative activation mechanism as yet unknown (*28*). Enzymes were expressed and purified under atmospheric conditions, and we assumed them to be largely oxidized. A 30 μM 5,5′-dithiobis-(2-nitrobenzoic acid) (DTNB) solution was added to reaction mixes before measurement to avoid the formation of unwanted disulfides with other proteins in clarified lysates. The final cuvette volume was brought to 600 μl. Figure S5 demonstrates that this does not alter CA activity or the response to RuBP. The rate of consumption of each measured isotope was determined at equilibrium before and after protein was added to the system and upon addition of RuBP when relevant. Activity measurements for *Cy*CsoSCA mutants were conducted as above all in the presence of 100 μM RuBP.

### Crystallization, data collection, and structure determination

Initial screening to determine crystallization conditions was performed at *Cy*CsoSCA concentrations of 10 mg/ml at 20/18°C in 96-well plates using the sitting drop vapor diffusion method and commercially available sparse matrix screens [ShotGun SG1 (Molecular Dimensions), PegIon (Hampton), Crystal Screen HT (Hampton), JCSG (Molecular Dimensions), PACT Premier (Molecular Dimensions), Index HT (Hampton), PegRx (Hampton), and Salt Rx (Hampton)]. In each case, 200-nl drops comprising 100 nl of protein solution and 100 nl of reservoir were prepared on hanging-drop seals using an NT8-Drop Setter robot (Formulatrix, Bedford, MA, USA) and equilibrated against 100 μl of reservoir solution. Further optimization was carried out in a 24-well hanging-drop vapor diffusion plate format under varying pH, precipitant, and protein concentration conditions. The final optimized crystallization conditions were at 0.2 M ammonium sulfate, 0.1 M bis-tris (pH 6.5), 21% polyethylene glycol 3350, and 15% ethylene glycol, seeded with crystals from the same conditions at a protein concentration of 7 mg/ml and an RuBP:CyCsoSCA molar concentration of 10:1. Crystals were cryoprotected with glycerol and flash-cooled in liquid nitrogen. X-ray diffraction data were collected at beamline MX2 at the Australian Synchrotron. Data were processed using XDS and Aimless, and molecular replacement was performed using Phaser. The ColabFold:AlphaFold2 used the MMseqs Google Colab notebook to generate a model of *Cy*CsoSCA for molecular replacement (*60*, *61*). Iterative cycles of manual model building and refinement were performed using Coot (*62*), ccp4 (*63*), and phenix.refine (*64*). TLS (Translation/Libration/Screw) parameter refinement was also used with TLS groups automatically selected by phenix.refine. Data collection and refinement statistics are provided in table S1. Structures were subsequently annotated and analyzed in PyMOL and PDBePISA (https://ebi.ac.uk/pdbe/pisa/).

### Molecular dynamics

The structure of *Cy*CsoSCA chain D reported here, thus the truncated *Cy*CsoSCA (fig. S1), was used for all simulations. For holoprotein preparation, chain D was used as is; otherwise, apoprotein starting structures were generated by deleting RuBP from the starting file. Automated structure refinement was conducted by the protein preparation wizard module of Maestro (Schrodinger, version 12.9.137 release 2021-3). This comprised assignment of bond order, addition of missing hydrogens, and generation of het states using the Epik program at a target pH of 7.4. The hydrogen bond network was subsequently optimized with the default parameters, and protonation states were assigned to residues at a pH of 7.4 using PROPKA. System builder was used to set up an orthorhombic box filled with TIP5P water models, the charge was neutralized with 18 Na^+^ ions, a buffer distance of 15 Å was used, and the OPLS4 force field was applied. A single run of the holoprotein and apoprotein with water model TIP4P was conducted for comparison (fig. S9). Short minimization was performed on the resultant structure of 1 ns. Simulations were performed at a total time of 300 ns at a time step of 50 ps in three replicates for each system at different initial random seeds. Resultant trajectories were analyzed using the simulation interaction diagram module in Schrodinger and using the MDTraj python package (*65*).

### Phylogenetic reconstruction

All CsoSCA sequences within the protein family (PF08936) were harvested from the Pfam database, including the primary query sequence WP_071778263.1. Duplicates were removed, and a local allvsall BLAST search (*66*) was conducted to generate a sequence similarity network of the sequences with an *e* value cutoff of 10e^−10^. This was used to identify classes within the family, and using incremental percent identity edge cutoffs in Cytoscape (version 3.9.1), clusters within these classes were examined. An additional 19 reviewed sequences of known β-CAs were appended to this database for comparison with the non-*cso* clade. Sequences without key catalytic sites and outliers were manually removed, and the remaining sequences were filtered using CD-HIT to remove redundancy to 90%, resulting in a final database of 504 sequences, and details of these, including accessions, are documented in the Supplementary Materials (19 reviewed β-CAs and 485 CsoSCA protein family sequences) (*67*). A multiple sequence alignment of these was constructed using MAFFT-LINSI (*68*). Columns with >90% gaps were removed using TrimAI through the pheylemon2 web server (http://phylemon2.bioinfo.cipf.es/) (*69*). Given the lack of conservation in the disordered N-terminal tail, there was little phylogenetic signal in this portion of the alignment; thus, it was removed during this editing process. This edited alignment was submitted to IQ-TREE (http://iqtree.cibiv.univie.ac.at/) for phylogenetic inference via ML (*70*, *71*). The ML sequence evolution model (WAG+F + I + G4) was selected as implemented in ModelFinder. Branch supports were measured by ultrafast bootstrap approximation and approximate likelihood ratio test, each conducted to 1000 replicates in IQ-TREE. Five independent replicates of tree search were conducted, one of which was selected due to high branch supports at key bifurcations. Trees were visualized and edited using iTOL (https://itol.embl.de/) (*72*). Sequence logos of alignments presented in the text were generated through WebLogo3 (https://weblogo.threeplusone.com/create.cgi) (*73*). To assess the genetic context of non-*cso* sequences, the genetic context was visualized and manually inspected through JGI IMG (https://img.jgi.doe.gov/) (*74*).

### COPASI modeling

Modeling of carboxysome function with and without RuBP-dependent CA activity was carried out using the COPASI biochemical modeling simulator as described previously (*35*). Modeling was carried out for a single *Cyanobium* carboxysome of 100 nm diameter, containing a Rubisco active-site concentration of 5.68 mM based on the average number of Rubisco holoenzymes per *Cyanobium* carboxysome previously reported at 224 (*29*) and the Rubisco catalytic parameters reported previously (*k*_catC_ = 9.4 s^−1^, *K*_MCO2_ = 169 μM, *K*_MO2_ = 1.4 mM, *k*_catO_ = 1.42 s^−1^, and *K*_MRuBP_ = 40 μM) (*29*, *35*). Modeling was carried out at 20 mM HCO_3_^−^, pH 8.0, and atmospheric O_2_ concentrations over an exponential range of RuBP concentrations from 0.1 μM to 5 mM, spanning the apparent *K*_M_ for CA activation (18 μM). Permeabilities were set to simulate a carboxysome, with CA activity confined to the Rubisco compartment (*35*). The model was run with either unmodified CA function or modified to be dependent on RuBP concentrations to replicate the observed RuBP response in Fig. 1. Specifically, the model modifies CA activity as a function of the deprotonated, and most abundant, form of RuBP within the carboxysome (RuBP^4−^).

Rate constant for the unmodified CA forward reaction (CO_2_ + H_2_O → HCO_3_^−^ + H^+^)

$$\text{CA factor}\times k_{1}\times {\left[{\text{CO}}_{2}\right]}_{c}$$

Rate constant for the unmodified CA backward reaction (HCO_3_^−^ + H^+^ → CO_2_ + H_2_O)

$$\text{CA factor}\times k_{2}\times {\left[{\text{HCO}}_{3}^{-}\right]}_{c}\times {\left[H^{+}\right]}_{c}$$

where the carboxysomal CA factor is set to 100,000, *k*_1_ is 0.05, *k*_2_ is 100, and [CO_2_]_c_, [HCO_3_^−^]_c_, and [H^+^]_c_ are the carboxysomal concentrations of CO_2_, HCO_3_^−^, and protons, respectively (*35*). Where CA function is not present in the model (in the external and unstirred compartments), CA factor is set to 1 (1 × the background rate of interconversion).

Carboxysomal CA function within the model was modified to be dependent on RuBP concentrations by multiplying each of the forward and backward rate constants by the following function

$$\frac{{{\left[\text{RuBP}\right]}_{c}}^{h}}{{K_{\text{half}}}^{h}+{{\left[\text{RuBP}\right]}_{c}}^{h}}$$

where [RuBP]_c_ is the carboxysomal concentration of RuBP^4−^, *h* is the Hill slope (set to 2.214), and *K*_half_ is the apparent *K*_MRuBP_ required to achieve half-maximal CA activity (18 μM). Both *h* and *K*_half_ were determined through Hill-reaction curve fitting of the RuBP response curve of *Cyanobium* CA in Fig. 1 using GraphPad Prism.

## Supplementary Materials

### This PDF file includes:

Supplementary Materials and Methods Tables S1 to S6 Figs. S1 to S15 Legends for data S1 to S3 References
