# Supplementary material for: Cyanobacterial α-carboxysome carbonic anhydrase is allosterically regulated by the Rubisco substrate RuBP
Source: Sci Adv. 2024 May 10;10(19):eadk7283. doi: 10.1126/sciadv.adk7283 (PMC11086599; doi:10.1126/sciadv.adk7283)
Supplement: Supplementary file 1 — Supplementary Materials and Methods Tables S1 to S6 Figs. S1 to S15 Legends for data S1 to S3 References [file sciadv.adk7283_sm.pdf]

Science Advances
AAAS

Supplementary Materials for

Cyanobacterial α-carboxysome carbonic anhydrase is allosterically regulated by the Rubisco substrate RuBP

Sacha B. Pulsford *et al.*

Corresponding author: Benedict M. Long, ben.long@newcastle.edu.au

Sci. Adv. **10**, eadk7283 (2024)
DOI: 10.1126/sciadv.adk7283

The PDF file includes:

Supplementary Materials and Methods
Tables S1 to S6
Figs. S1 to S15
Legends for data S1 to S3
References

Other Supplementary Material for this manuscript includes the following:

Data S1 to S3

Supplemental Materials and Methods

Ion inhibition assays

To determine the effect of sulfate ions on CsoSCA activity, the standard buffer described above was substituted for either EPPS only (50mM EPPS-NaOH pH 7.8), 50mM MgCl2 (50mM EPPS-NaOH pH 7.8, 50mM MgCl2), 20mM MgSO4 (50mM EPPS-NaOH pH 7.8, 20mM MgSO4) and 50mM MgSO4 (50mM EPPS-NaOH pH 7.8, 50mM MgSO4). Measurements for *Cy*CsoSCA were taken in the presence of 100uM RuBP, given no dependency on RuBP was observed for *Hn*CsoSCA it was not added to these activity conditions.

Analytical size exclusion chromatography

A HiLoad 16/600 Superose 6pg preparative size exclusion chromatography (SEC) column was calibrated using the Cytiva Gel Filtration Calibration HMW kit (product code 28403842) as per manual specifications (28951560 AG). The partition coefficient (Kav) of each protein was used to construct a calibration curve of Kav versus log(molecular mass). This was calculated using the equation Kav = (ve – vo)/(vc – vo), where ve is the elution volume, vo is the column void volume, and vc is the geometric column volume. These were used for comparison of elution columns and estimation of theoretical molecular masses.

Native PAGE

Protein samples were stored in Native Gel-Loading buffer (2.5 x TBE, 50% Glycerol, 0.1% Bromophenol blue) and loaded on 4–20% Mini-PROTEAN TGX Stain-free polyacrylamide gels (Bio-Rad, Cat. No. 4568096). Proteins were separated at 90 V for 90 minutes at 4°C in native running buffer (25 mM Tris [pH 8.3], 50 mM glycine). NativeMark protein standards were used for molecular weight determination (Invitrogen, LOT 1739010). To visualise proteins, gels were stained with Coomassie Blue dye (BioRad, USA). To determine how essential the observed zinc ions in the *Cy*CsoSCA structure are for hexamer formation, the protein was treated with a range of conditions to interrupt zinc binding, the oligomeric state of resulting samples was then assessed by Native PAGE. CyCsoSCA was dialysed with 2mM 1,10-phenanthroline, a strong chelating agent, for 24 hours according to previous publications[76]. Additionally, protein samples were incubated in SEC buffer at a pH of 3.5, 4, 5, 6, 7, or 8 for 1 hour. All samples were stored in Native Gel-Loading buffer (2.5 x TBE, 50% Glycerol, 0.1% Bromophenol blue) and loaded on 4–20% Mini-PROTEAN TGX Stain-free polyacrylamide gels (Bio-Rad, Cat. No. 4568096). Proteins were separated at 90 V for 90

minutes at 4°C in native running buffer (25 mM Tris [pH 8.3], 50 mM glycine). NativeMark protein standards were used for molecular weight determination (Invitrogen, LOT 1739010). To visualise proteins, gels were stained with Coomassie Blue dye (BioRad, USA).

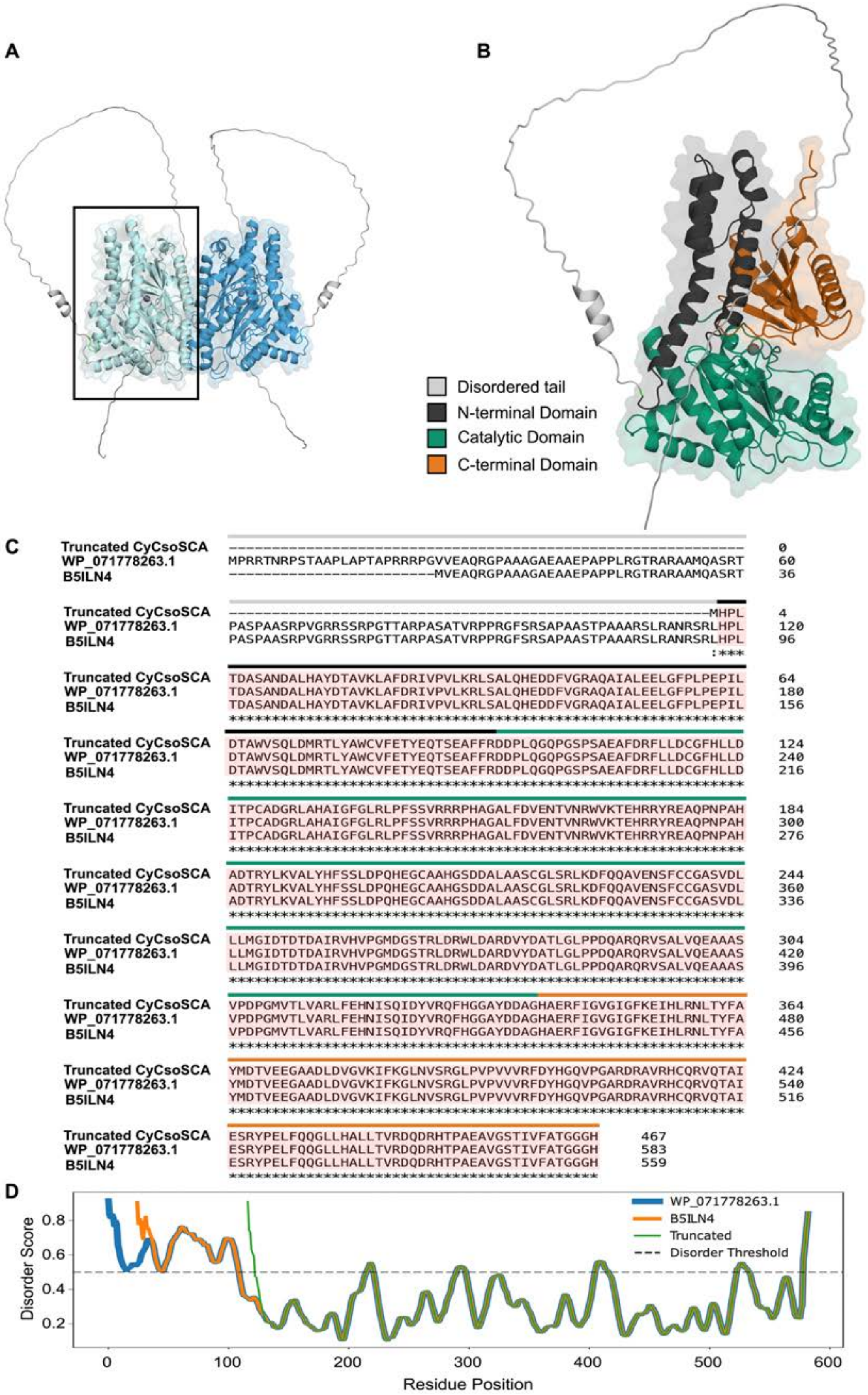

Figure S1. **CsoSCA homologues contain an extended disordered tail that has been truncated in this study to mitigate aggregation.** The N-terminal disordered tail binds to Rubisco to enable CsoSCA encapsulation during α-carboxysome biogenesis[30]. In the

automated annotation of *Cy*CsoSCA, an internal start site within the disordered site was mistakenly identified, leading to different sequences of variable lengths attributed to *Cy*CSoSCA in Uniprot (B5ILN4) and NCBI (WP_071778263.1) databases. The sequence WP_071778263.1 is the complete, 'full-length' sequence. **A** AlphaFold2 multimer model of the full-length *Cy*CsoSCA (WP_071778263.1) homodimer, a black box highlights a monomeric unit. The AlphaFold2 model was used in lieu of the available crystal structure (PDB ID: 8thm) to demonstrate how the flexible disordered tail may arrange around the globular domains in such a complex. Each monomer is coloured a different shade of blue, the disordered tail is shown in cartoon form in light grey. The catalytic zinc was modelled in with reference to the available structure (PDB ID: 8thm) and is shown as a grey sphere. **B** The full-length monomer coloured by domain as indicated. The catalytic zinc was modelled in with reference to the available structure (PDB ID: 8thm) and is shown as a grey sphere. **C** A multiple sequence alignment of the full-length *Cy*CsoSCA (WP_0717782663.1), the sequence mis-annotated at an internal start site (B5ILN4), and the truncated form ('Truncated CyCsoSCA') used in this study in all analyses lacks the extended disordered region (116 residues from the N-terminus removed). The globular region of the protein is highlighted with a red box, the Disordered tail, N-terminal domain, Catalytic domain and the C-terminal domain are indicated with lines coloured as in panel B. **D** DisEMBL disordered prediction scores for the three sequences aligned in panel C with a disorder threshold marked at disorder score 0.5.

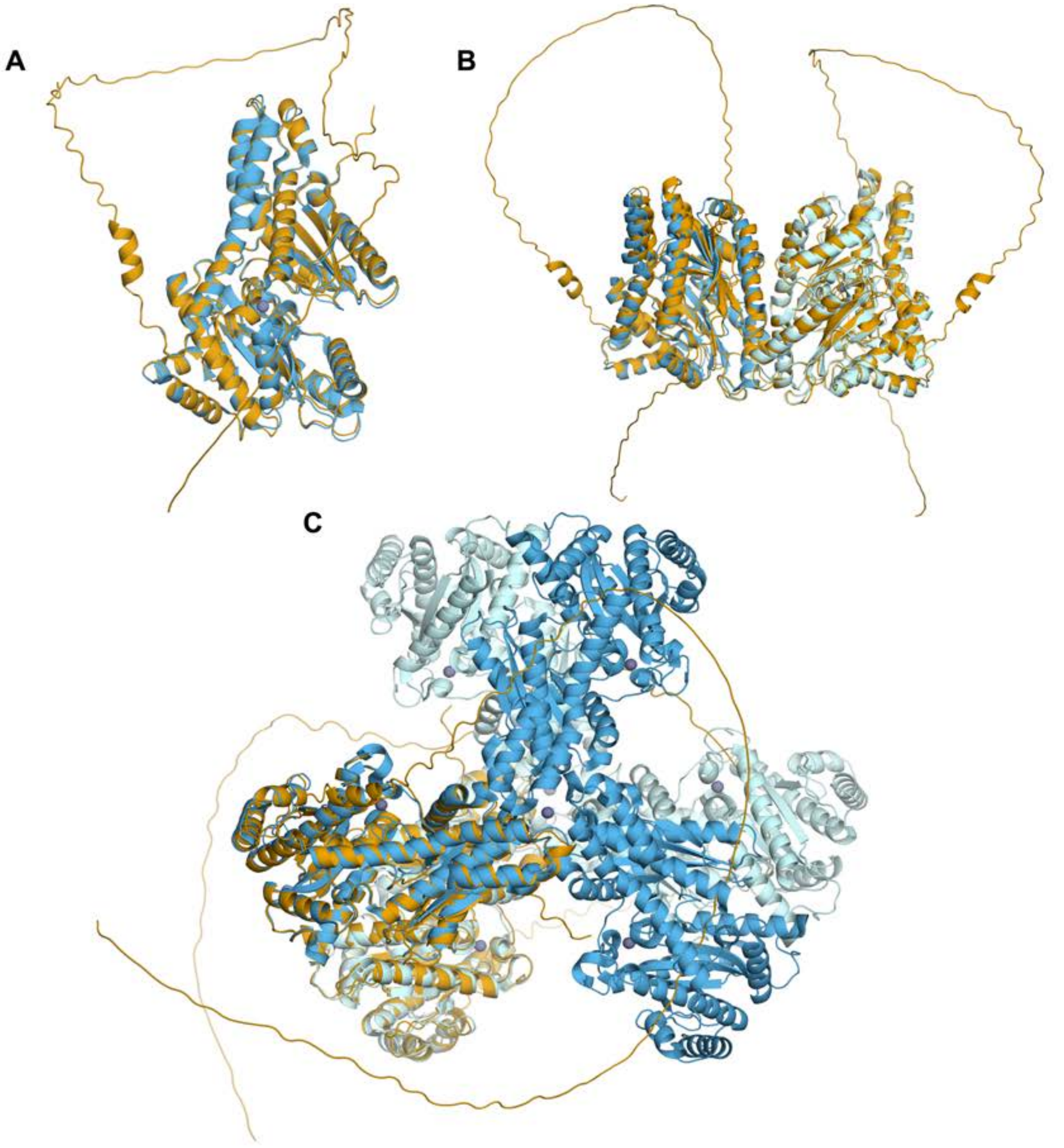

Figure S2 Comparison of αFold2 models of full length *Cy*CsoSCA to the crystalised truncated form. A Alphafold2 monomer (orange) and chain A of 8thm structures aligned (Cα RMSD 0.541 A). Catalytic zinc included for reference as a grey sphere, no notable deviations noted. **B** AlphaFold2 multimer model of full length *Cy*CsoSCA sequences in a dimer aligned to 8thm chain A-B dimer (Cα RMSD 1.214). No major structural deviations evident between model and crystal structure. **C** The modelled dimer (orange) with the extended disordered N-terminal aligned with the complete *Cy*CsoSCA hexamer observed in the crystal structure. The disordered termini, while long is flexible and thus should not sterically hinder the formation of the hexameric complex. One can imagine the increased density of disordered strands due to hexamer assembly may notably increase the propensity for aggregation relative to solutions in which the dimer dominates.

Table S1. Data collection and refinement details for the *Cy*CsoSCA structure presented in this work.

**PDB ID**	**8THM**
**Data collection**	
Space group	P 21 21 21
Cell dimensions	
a, b, c (Å)	104.314, 181.848, 190.057
a, b, g (°)	90, 90, 90
Resolution (Å)	131.392-2.300
R_{merge}	
Within I+/I- (overall, inner, outer)	0.250, 0.052, 4.833
All I+ and I- (overall, inner, outer)	0.260, 0.0.54, 5.093
R_{pim}	
Within I+/I- (overall, inner, outer)	0.107, 0.022, 2.031
All I+ and I- (overall, inner, outer)	0.077, 0.017,1.468
I/σI	8.1, 32.3, 0.7
$CC_{1/2}$	0.997, 0.999, 0.297
Completeness (%)	100.0, 98.3, 100.0
Redundancy (multiplicity)	12.3,10.4,12.7
**Refinement**	
Resolution (Å)	47.514-2.3 (2.382-2.300)
No. reflections	160413 (15835)
R_{work}/R_{free}	0.1827/0.2392
No. atoms	23052
Protein	21974
Ligand/ion	282
Water	796
B-factors (overall)	Mean(55.7), max(129.2), min(31.1)
Protein	Mean(55.7) max(129.2), min(31.1)
Ligand/ion	Mean(61.3), max(95.9), min(40.2)
Water	Mean(55.2), max(95.3), min(36.3)
R.m.s. deviations	
Bond lengths (Å)	0.0090
Bond angles (Å)	1.24

Table S2. Details of the RuBP binding site for each chain of the *Cy*CsoSCA structure presented in this work.

Chain	PO_4 head 1	C-chain	PO_4 head 2
**A**	R266 (sidechain and backbone), K469, water	4xwaters, K469	Water, R560, D517
**B**	R266(sidechain and backbone), water	4xwaters, R265, D517	R265
**C**	K469	3xwaters, K469	R560, water
**D**	R266, H356, K469 (backbone)	K469, R149	R560, K469, D517
**E**	R266, water	R265, K469	R560, D517, K469
**F**	2xwaters, K469	R149, 2xwaters, R560	R560, 2xwaters

Table S3. Residues involved in binding sulfate ions within each chain of the *Cy*CsoSCA structure presented here.

Chain	SO_4 no.1	SO_4 no. 2	SO_4 no. 3
**A**	R265, 2xwaters, N278	2xwater, R267, N278, K307, H269	R266, water, 2 bbone interactions
**B**	R265, 3xwater, R282	2xwater, R267, N278, H269, R282	
**C**	R265, K285, R282, 2xwaters, R151	2xwaters, H269, N278, R267, R282	
**D**	R265, R282, K285	H269, R282, N278, R267, water	R266, F468 (backbone), I471 (backbone), water
**E**	R265, K285, R282	R282, N278, R267, H269, water	2xwater, R266, F468 (backbone)
**F**	K285, 2xwaters, R265, R282	R282, 2xwaters, H269, R267, N278	R266, 2xwaters, F468 (backbone), I471 (backbone)

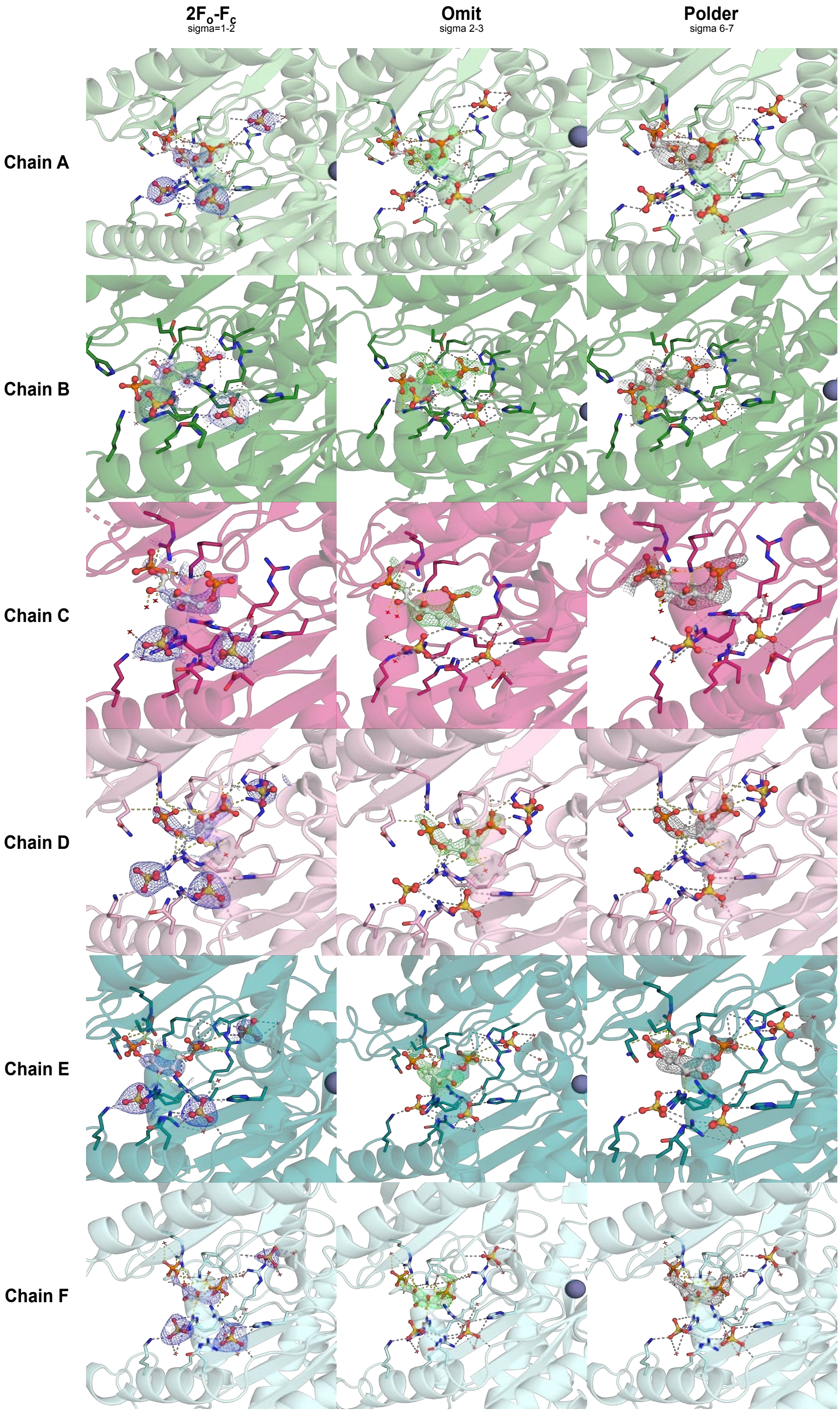

Figure S3 Density maps of RuBP in each monomer in the *Cy*CsoSCA structure characterised in the main text. At least two sulfate ions are observed at this site in all monomers from the crystallisation solvent. Sulfate ions have been observed bound to β-CA oligomeric interfaces, though rarely at pockets buried so deep as observed here[27].

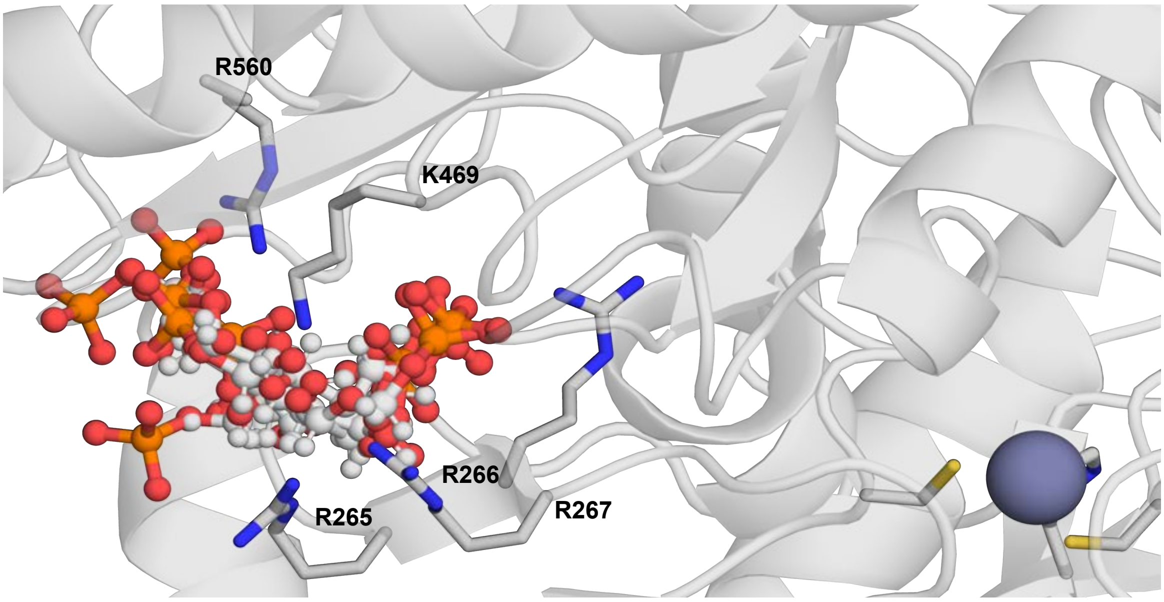

Figure S4. RuBP molecules in each monomer overlayed. The ligand is shown in ball and stick representation and coloured by element. Key sidechains are shown in stick representation and annotated. For perspective, the overlayed ligands have been situated in chain D (cartoon representation) with the zinc active site shown as a grey sphere.

Table S4 Kinetics data for wild type *Cy*CsoSCA, *Hn*CsoSCA and plotted in Figure 1D and Figure 3D of the main text. Continued on the following page.

Time	CyCsoSCA	HnCsoSCA	T477A	K469D	H472Q	I466D	H519Q	Loop+	H436N	Loop-
0.0	1.79 ± 0.185	3.55 ± 0.076	1.88 ± 0.015	1.84 ± 0.015	1.88 ± 0.026	1.83 ± 0.015	2.03 ± 0.036	2.05 ± 0.021	1.99 ± 0.065	1.95 ± 0.225
1.0	1.78 ± 0.174	3.57 ± 0.11	1.89 ± 0.021	1.84 ± 0.02	1.88 ± 0.032	1.83 ± 0.015	2.04 ± 0.025	2.05 ± 0.021	1.99 ± 0.05	1.95 ± 0.212
2.0	1.77 ± 0.186	3.58 ± 0.122	1.89 ± 0.017	1.84 ± 0.03	1.88 ± 0.021	1.84 ± 0.015	2.04 ± 0.035	2.05 ± 0.02	2.0 ± 0.045	1.96 ± 0.201
3.0	1.77 ± 0.164	3.57 ± 0.135	1.9 ± 0.021	1.84 ± 0.023	1.88 ± 0.035	1.85 ± 0.015	2.04 ± 0.015	2.06 ± 0.03	1.99 ± 0.05	1.96 ± 0.201
4.0	1.79 ± 0.178	3.57 ± 0.135	1.89 ± 0.015	1.84 ± 0.031	1.88 ± 0.032	1.86 ± 0.015	2.04 ± 0.023	2.06 ± 0.031	2.01 ± 0.056	1.97 ± 0.184
5.0	1.79 ± 0.166	3.57 ± 0.156	1.89 ± 0.021	1.84 ± 0.025	1.88 ± 0.025	1.86 ± 0.015	2.0 ± 0.055	2.06 ± 0.031	2.01 ± 0.055	1.97 ± 0.178
6.0	1.8 ± 0.155	3.57 ± 0.125	1.9 ± 0.026	1.84 ± 0.017	1.88 ± 0.031	1.87 ± 0.006	2.05 ± 0.017	2.06 ± 0.035	2.02 ± 0.05	1.98 ± 0.166
7.0	1.81 ± 0.161	3.58 ± 0.151	1.9 ± 0.031	1.83 ± 0.012	1.87 ± 0.025	1.88 ± 0.015	2.05 ± 0.023	2.07 ± 0.04	2.01 ± 0.045	1.99 ± 0.155
8.0	1.81 ± 0.159	3.57 ± 0.115	1.9 ± 0.021	1.84 ± 0.015	1.88 ± 0.036	1.88 ± 0.02	2.05 ± 0.029	2.07 ± 0.042	2.02 ± 0.045	1.99 ± 0.147
9.0	1.81 ± 0.15	3.59 ± 0.16	1.91 ± 0.023	1.84 ± 0.015	1.88 ± 0.035	1.89 ± 0.021	2.04 ± 0.021	2.07 ± 0.042	2.03 ± 0.05	2.0 ± 0.181
10.0	1.82 ± 0.139	3.61 ± 0.199	1.96 ± 0.06	1.9 ± 0.044	1.88 ± 0.032	1.89 ± 0.015	2.05 ± 0.015	2.08 ± 0.04	2.03 ± 0.045	2.01 ± 0.147
11.0	1.82 ± 0.139	3.85 ± 0.35	2.12 ± 0.208	2.07 ± 0.13	1.91 ± 0.036	1.9 ± 0.015	2.03 ± 0.035	2.08 ± 0.038	2.04 ± 0.04	2.01 ± 0.151
12.0	1.82 ± 0.145	4.51 ± 0.835	2.48 ± 0.495	2.44 ± 0.257	1.98 ± 0.079	1.91 ± 0.015	2.05 ± 0.025	2.08 ± 0.042	2.04 ± 0.04	2.02 ± 0.14
13.0	1.82 ± 0.142	5.92 ± 1.61	3.17 ± 0.881	3.03 ± 0.41	2.1 ± 0.15	1.9 ± 0.021	2.05 ± 0.021	2.08 ± 0.032	2.03 ± 0.035	2.02 ± 0.144
14.0	1.81 ± 0.146	8.29 ± 2.518	4.2 ± 1.38	3.86 ± 0.56	2.28 ± 0.223	1.91 ± 0.021	2.06 ± 0.026	2.08 ± 0.035	2.04 ± 0.042	2.02 ± 0.131
15.0	1.81 ± 0.129	11.78 ± 3.478	5.6 ± 1.989	4.93 ± 0.719	2.54 ± 0.289	1.91 ± 0.023	2.06 ± 0.03	2.08 ± 0.032	2.04 ± 0.035	2.03 ± 0.123
16.0	1.82 ± 0.137	16.34 ± 4.435	7.33 ± 2.645	6.22 ± 0.849	2.85 ± 0.352	1.91 ± 0.02	2.08 ± 0.031	2.09 ± 0.038	2.04 ± 0.045	2.03 ± 0.121
17.0	1.82 ± 0.137	21.9 ± 5.218	9.37 ± 3.355	7.69 ± 0.953	3.22 ± 0.406	1.91 ± 0.017	2.05 ± 0.031	2.09 ± 0.038	2.04 ± 0.038	2.03 ± 0.117
18.0	1.83 ± 0.133	28.3 ± 5.851	11.66 ± 4.07	9.26 ± 1.042	3.62 ± 0.445	1.92 ± 0.02	2.08 ± 0.049	2.09 ± 0.04	2.04 ± 0.025	2.04 ± 0.112
19.0	1.83 ± 0.142	35.29 ± 6.182	14.12 ± 4.721	10.87 ± 1.074	4.06 ± 0.465	1.92 ± 0.017	2.09 ± 0.046	2.1 ± 0.044	2.04 ± 0.021	2.04 ± 0.106
20.0	1.83 ± 0.142	42.56 ± 6.245	16.69 ± 5.211	12.46 ± 1.074	4.5 ± 0.466	1.92 ± 0.015	2.11 ± 0.04	2.1 ± 0.042	2.05 ± 0.021	2.03 ± 0.096
21.0	1.83 ± 0.137	49.73 ± 5.964	19.22 ± 5.52	13.91 ± 1.008	4.9 ± 0.438	1.92 ± 0.006	2.12 ± 0.055	2.11 ± 0.047	2.04 ± 0.015	2.04 ± 0.091
22.0	1.84 ± 0.137	56.48 ± 5.395	21.62 ± 5.597	15.19 ± 0.943	5.29 ± 0.377	1.93 ± 0.006	2.12 ± 0.04	2.1 ± 0.04	2.04 ± 0.01	2.04 ± 0.089
23.0	1.84 ± 0.147	62.49 ± 4.629	23.92 ± 5.455	16.3 ± 0.88	5.62 ± 0.322	1.93 ± 0.006	2.13 ± 0.05	2.11 ± 0.038	2.05 ± 0.012	2.05 ± 0.085
24.0	1.84 ± 0.147	67.69 ± 3.858	26.0 ± 5.166	17.27 ± 0.806	5.9 ± 0.296	1.92 ± 0.01	2.16 ± 0.035	2.11 ± 0.052	2.05 ± 0.017	2.05 ± 0.081
25.0	1.84 ± 0.144	72.15 ± 3.232	27.93 ± 4.731	18.09 ± 0.755	6.15 ± 0.269	1.93 ± 0.006	2.15 ± 0.031	2.12 ± 0.044	2.05 ± 0.017	2.06 ± 0.079
26.0	1.86 ± 0.147	76.04 ± 2.691	29.72 ± 4.195	18.81 ± 0.707	6.37 ± 0.266	1.93 ± 0.0	2.18 ± 0.032	2.11 ± 0.032	2.05 ± 0.012	2.06 ± 0.078
27.0	1.87 ± 0.133	79.29 ± 2.17	31.34 ± 3.671	19.41 ± 0.668	6.57 ± 0.266	1.93 ± 0.012	2.17 ± 0.026	2.12 ± 0.046	2.05 ± 0.01	2.06 ± 0.07
28.0	1.86 ± 0.15	82.07 ± 1.806	32.84 ± 3.151	19.96 ± 0.647	6.71 ± 0.266	1.93 ± 0.015	2.18 ± 0.017	2.12 ± 0.044	2.06 ± 0.012	2.07 ± 0.075
29.0	1.88 ± 0.156	84.48 ± 1.51	34.16 ± 2.742	20.42 ± 0.627	6.86 ± 0.271	1.94 ± 0.006	2.19 ± 0.015	2.12 ± 0.044	2.06 ± 0.012	2.06 ± 0.078
30.0	1.88 ± 0.153	86.53 ± 1.255	35.33 ± 2.506	20.85 ± 0.611	7.0 ± 0.282	1.95 ± 0.017	2.19 ± 0.025	2.12 ± 0.046	2.06 ± 0.015	2.08 ± 0.049
31.0	1.87 ± 0.162	88.3 ± 1.033	36.36 ± 2.35	21.21 ± 0.597	7.1 ± 0.289	1.94 ± 0.0	2.21 ± 0.021	2.12 ± 0.04	2.05 ± 0.015	2.08 ± 0.05
32.0	1.89 ± 0.168	89.86 ± 0.804	37.27 ± 2.325	21.52 ± 0.612	7.22 ± 0.31	1.94 ± 0.0	2.21 ± 0.015	2.11 ± 0.025	2.06 ± 0.015	2.07 ± 0.025
33.0	1.88 ± 0.162	91.22 ± 0.665	38.08 ± 2.346	21.81 ± 0.584	7.3 ± 0.343	1.95 ± 0.0	2.22 ± 0.025	2.13 ± 0.044	2.06 ± 0.021	2.09 ± 0.046
34.0	1.89 ± 0.165	92.4 ± 0.53	38.76 ± 2.512	22.16 ± 0.712	7.42 ± 0.327	1.95 ± 0.01	2.22 ± 0.025	2.14 ± 0.044	2.06 ± 0.021	2.1 ± 0.053
35.0	1.9 ± 0.177	93.48 ± 0.42	39.43 ± 2.552	22.44 ± 0.795	7.52 ± 0.376	1.95 ± 0.01	2.23 ± 0.025	2.14 ± 0.053	2.05 ± 0.01	2.11 ± 0.053
36.0	1.89 ± 0.173	94.38 ± 0.354	39.98 ± 2.645	22.7 ± 0.862	7.63 ± 0.417	1.96 ± 0.021	2.23 ± 0.04	2.14 ± 0.046	2.07 ± 0.012	2.12 ± 0.044
37.0	1.9 ± 0.182	95.16 ± 0.288	40.49 ± 2.752	22.87 ± 0.852	7.71 ± 0.422	1.96 ± 0.015	2.22 ± 0.036	2.14 ± 0.051	2.06 ± 0.015	2.13 ± 0.064
38.0	1.89 ± 0.182	95.8 ± 0.346	40.91 ± 2.863	23.13 ± 0.958	7.8 ± 0.446	1.96 ± 0.012	2.23 ± 0.032	2.08 ± 0.05	2.06 ± 0.015	2.13 ± 0.049
39.0	1.89 ± 0.18	96.38 ± 0.401	41.29 ± 2.965	23.32 ± 0.997	7.91 ± 0.477	1.96 ± 0.006	2.21 ± 0.047	2.15 ± 0.079	2.05 ± 0.006	2.13 ± 0.068
40.0	1.88 ± 0.159	96.91 ± 0.37	41.64 ± 3.078	23.54 ± 1.129	7.99 ± 0.493	1.97 ± 0.012	2.23 ± 0.035	2.15 ± 0.079	2.06 ± 0.0	2.15 ± 0.049
41.0	1.9 ± 0.187	97.3 ± 0.395	41.96 ± 3.154	23.74 ± 1.247	8.05 ± 0.547	1.96 ± 0.006	2.23 ± 0.04	2.15 ± 0.081	2.07 ± 0.006	2.16 ± 0.042
42.0	1.9 ± 0.185	97.73 ± 0.457	42.25 ± 3.24	23.89 ± 1.266	8.13 ± 0.562	1.96 ± 0.006	2.23 ± 0.032	2.16 ± 0.074	2.07 ± 0.025	2.17 ± 0.042
43.0	1.9 ± 0.173	98.02 ± 0.457	42.54 ± 3.29	24.07 ± 1.243	8.19 ± 0.567	1.96 ± 0.015	2.23 ± 0.035	2.16 ± 0.074	2.07 ± 0.012	2.18 ± 0.05
44.0	1.89 ± 0.182	98.3 ± 0.474	42.76 ± 3.365	24.25 ± 1.244	8.23 ± 0.566	1.97 ± 0.015	2.22 ± 0.032	2.14 ± 0.068	2.08 ± 0.012	2.16 ± 0.026
45.0	1.89 ± 0.184	98.5 ± 0.523	43.01 ± 3.402	24.42 ± 1.23	8.27 ± 0.601	1.97 ± 0.025	2.23 ± 0.038	2.16 ± 0.064	2.08 ± 0.01	2.17 ± 0.038
46.0	1.9 ± 0.169	98.7 ± 0.58	43.22 ± 3.411	24.55 ± 1.213	8.32 ± 0.593	1.97 ± 0.021	2.22 ± 0.029	2.16 ± 0.061	2.08 ± 0.012	2.18 ± 0.045
47.0	1.89 ± 0.18	98.86 ± 0.637	43.4 ± 3.457	24.73 ± 1.26	8.37 ± 0.585	1.96 ± 0.012	2.23 ± 0.032	2.16 ± 0.061	2.09 ± 0.01	2.18 ± 0.04
48.0	1.9 ± 0.185	99.05 ± 0.622	43.57 ± 3.506	24.83 ± 1.26	8.42 ± 0.607	1.97 ± 0.017	2.24 ± 0.031	2.16 ± 0.061	2.1 ± 0.006	2.18 ± 0.04
49.0	1.9 ± 0.182	99.21 ± 0.677	43.73 ± 3.515	24.96 ± 1.245	8.44 ± 0.617	1.97 ± 0.023	2.22 ± 0.031	2.17 ± 0.064	2.1 ± 0.01	2.19 ± 0.03
50.0	1.91 ± 0.178	99.29 ± 0.637	43.86 ± 3.552	25.11 ± 1.251	8.45 ± 0.596	1.97 ± 0.026	2.25 ± 0.056	2.1 ± 0.064	2.11 ± 0.012	2.19 ± 0.036
51.0	1.91 ± 0.171	99.38 ± 0.668	43.99 ± 3.59	25.23 ± 1.253	8.47 ± 0.61	1.97 ± 0.015	2.24 ± 0.049	2.16 ± 0.038	2.12 ± 0.023	2.18 ± 0.038
52.0	1.9 ± 0.17	99.46 ± 0.703	44.09 ± 3.624	25.34 ± 1.262	8.5 ± 0.59	1.97 ± 0.026	2.26 ± 0.05	2.16 ± 0.035	2.12 ± 0.01	2.19 ± 0.035
53.0	1.9 ± 0.17	99.36 ± 0.694	44.2 ± 3.675	25.52 ± 1.241	8.52 ± 0.585	1.97 ± 0.0	2.26 ± 0.05	2.16 ± 0.046	2.12 ± 0.006	2.19 ± 0.02
54.0	1.9 ± 0.154	99.42 ± 0.685	44.27 ± 3.722	25.67 ± 1.215	8.53 ± 0.595	1.98 ± 0.01	2.27 ± 0.057	2.15 ± 0.04	2.13 ± 0.01	2.19 ± 0.025
55.0	1.9 ± 0.168	99.42 ± 0.692	44.39 ± 3.755	25.75 ± 1.177	8.55 ± 0.575	1.98 ± 0.0	2.27 ± 0.066	2.15 ± 0.04	2.13 ± 0.006	2.19 ± 0.032
56.0	1.89 ± 0.171	99.45 ± 0.688	44.49 ± 3.781	25.85 ± 1.123	8.58 ± 0.584	1.98 ± 0.006	2.26 ± 0.061	2.16 ± 0.035	2.12 ± 0.021	2.2 ± 0.036
57.0	1.91 ± 0.163	99.41 ± 0.745	44.59 ± 3.818	25.96 ± 1.1	8.62 ± 0.558	1.97 ± 0.006	2.28 ± 0.066	2.16 ± 0.04	2.13 ± 0.006	2.19 ± 0.025
58.0	1.92 ± 0.18	99.43 ± 0.695	44.68 ± 3.832	26.0 ± 1.077	8.65 ± 0.528	1.98 ± 0.012	2.27 ± 0.055	2.15 ± 0.04	2.14 ± 0.006	2.19 ± 0.035
59.0	1.93 ± 0.172	99.44 ± 0.686	44.74 ± 3.815	26.06 ± 1.043	8.66 ± 0.497	1.98 ± 0.012	2.25 ± 0.025	2.16 ± 0.04	2.13 ± 0.025	2.2 ± 0.04
60.0	1.92 ± 0.17	99.52 ± 0.69	44.82 ± 3.863	26.15 ± 1.03	8.69 ± 0.488	1.97 ± 0.017	2.27 ± 0.046	2.18 ± 0.04	2.13 ± 0.01	2.2 ± 0.035
61.0	1.93 ± 0.199	99.56 ± 0.629	44.95 ± 3.881	26.2 ± 1.006	8.74 ± 0.479	1.97 ± 0.012	2.26 ± 0.015	2.22 ± 0.036	2.12 ± 0.01	2.21 ± 0.025
62.0	2.03 ± 0.333	99.49 ± 0.56	45.04 ± 3.94	26.29 ± 0.947	8.76 ± 0.453	1.97 ± 0.006	2.28 ± 0.035	2.31 ± 0.035	2.13 ± 0.006	2.24 ± 0.025
63.0	2.44 ± 0.74	99.57 ± 0.417	45.1 ± 3.939	26.37 ± 0.908	8.77 ± 0.446	1.97 ± 0.0	2.27 ± 0.036	2.46 ± 0.029	2.13 ± 0.021	2.34 ± 0.146

64.0	3.5 ± 1.375	99.56 ± 0.259	45.13 ± 3.888	26.41 ± 0.866	8.81 ± 0.446	1.97 ± 0.006	2.28 ± 0.029	2.67 ± 0.044	2.13 ± 0.02	2.56 ± 0.321
65.0	5.53 ± 2.132	99.59 ± 0.173	45.21 ± 3.82	26.48 ± 0.838	8.86 ± 0.437	1.97 ± 0.015	2.27 ± 0.035	2.92 ± 0.052	2.16 ± 0.053	2.91 ± 0.545
66.0	8.69 ± 2.942	99.65 ± 0.111	45.36 ± 3.669	26.59 ± 0.827	8.87 ± 0.409	1.98 ± 0.021	2.28 ± 0.038	3.23 ± 0.046	2.28 ± 0.275	3.45 ± 0.83
67.0	13.03 ± 3.74	99.68 ± 0.055	45.66 ± 3.441	26.72 ± 0.864	8.88 ± 0.381	1.98 ± 0.02	2.34 ± 0.015	3.56 ± 0.035	2.6 ± 0.826	4.16 ± 1.1
68.0	18.49 ± 4.404	99.77 ± 0.04	46.13 ± 3.228	26.87 ± 0.919	9.01 ± 0.332	1.98 ± 0.02	2.64 ± 0.106	3.93 ± 0.006	3.18 ± 1.784	5.05 ± 1.335
69.0	24.92 ± 4.952	99.81 ± 0.023	46.84 ± 3.127	27.09 ± 0.997	9.17 ± 0.232	1.98 ± 0.015	3.36 ± 0.281	4.27 ± 0.031	4.12 ± 3.127	6.06 ± 1.53
70.0	32.04 ± 5.311	99.88 ± 0.095	47.78 ± 3.124	27.32 ± 1.084	9.41 ± 0.083	1.98 ± 0.015	4.7 ± 0.491	4.6 ± 0.067	5.57 ± 4.719	7.19 ± 1.63
71.0	39.54 ± 5.373	99.83 ± 0.118	49.03 ± 3.311	27.59 ± 1.186	9.78 ± 0.096	1.99 ± 0.025	6.67 ± 0.772	4.89 ± 0.106	7.63 ± 6.391	8.35 ± 1.665
72.0	47.11 ± 5.179	99.84 ± 0.095	50.43 ± 3.614	27.9 ± 1.278	10.3 ± 0.243	1.99 ± 0.025	9.35 ± 1.008	5.14 ± 0.159	10.4 ± 7.94	9.5 ± 1.611
73.0	54.28 ± 4.72	99.82 ± 0.075	52.07 ± 3.951	28.29 ± 1.371	10.91 ± 0.323	1.99 ± 0.026	12.62 ± 1.327	5.35 ± 0.186	13.87 ± 9.195	10.58 ± 1.469
74.0	60.78 ± 4.091	99.89 ± 0.116	53.84 ± 4.224	28.66 ± 1.476	11.71 ± 0.516	1.97 ± 0.015	16.43 ± 1.62	5.51 ± 0.219	17.89 ± 10.053	11.55 ± 1.266
75.0	66.41 ± 3.448	99.89 ± 0.122	55.65 ± 4.431	29.04 ± 1.511	12.52 ± 0.592	2.0 ± 0.049	20.63 ± 1.896	5.65 ± 0.234	22.36 ± 10.418	12.38 ± 1.06
76.0	71.24 ± 2.889	99.79 ± 0.121	57.47 ± 4.544	29.42 ± 1.521	13.39 ± 0.599	2.01 ± 0.052	25.02 ± 2.243	5.77 ± 0.243	27.02 ± 10.191	13.09 ± 0.91
77.0	75.28 ± 2.449	99.72 ± 0.146	59.16 ± 4.637	29.79 ± 1.466	14.27 ± 0.565	2.01 ± 0.049	29.39 ± 2.541	5.87 ± 0.262	31.66 ± 9.416	13.67 ± 0.764
78.0	78.82 ± 2.08	99.65 ± 0.258	60.75 ± 4.672	30.15 ± 1.415	15.13 ± 0.483	2.01 ± 0.053	33.48 ± 2.839	5.97 ± 0.276	36.13 ± 8.228	14.16 ± 0.709
79.0	81.8 ± 1.744	99.59 ± 0.344	62.14 ± 4.678	30.47 ± 1.311	15.91 ± 0.319	2.02 ± 0.055	37.15 ± 3.13	6.04 ± 0.288	40.26 ± 6.746	14.55 ± 0.681
80.0	84.31 ± 1.502	99.5 ± 0.355	63.4 ± 4.677	30.82 ± 1.229	16.6 ± 0.24	2.02 ± 0.047	40.3 ± 3.414	6.13 ± 0.302	43.92 ± 5.229	14.88 ± 0.662
81.0	86.56 ± 1.248	99.5 ± 0.407	64.47 ± 4.712	31.12 ± 1.185	17.19 ± 0.169	2.02 ± 0.057	42.97 ± 3.651	6.19 ± 0.316	47.0 ± 3.874	15.16 ± 0.671
82.0	88.52 ± 1.075	99.45 ± 0.444	65.37 ± 4.73	31.38 ± 1.092	17.69 ± 0.231	2.02 ± 0.061	45.3 ± 3.879	6.23 ± 0.325	49.52 ± 2.744	15.37 ± 0.682
83.0	90.24 ± 0.898	99.38 ± 0.503	66.2 ± 4.781	31.63 ± 1.057	18.17 ± 0.269	2.01 ± 0.053	47.24 ± 4.067	6.28 ± 0.33	51.52 ± 1.958	15.57 ± 0.704
84.0	91.75 ± 0.739	99.43 ± 0.56	66.84 ± 4.783	31.83 ± 0.991	18.55 ± 0.318	2.01 ± 0.051	48.92 ± 4.22	6.33 ± 0.336	53.12 ± 1.456	15.71 ± 0.7
85.0	93.03 ± 0.595	99.4 ± 0.659	67.4 ± 4.901	32.02 ± 0.947	18.84 ± 0.453	2.01 ± 0.06	50.37 ± 4.369	6.37 ± 0.34	54.43 ± 1.255	15.84 ± 0.73
86.0	94.19 ± 0.451	99.46 ± 0.699	67.91 ± 4.951	32.17 ± 0.911	19.17 ± 0.453	1.98 ± 0.026	51.65 ± 4.498	6.39 ± 0.345	55.51 ± 1.277	15.94 ± 0.719
87.0	95.2 ± 0.34	99.45 ± 0.703	68.31 ± 4.92	32.33 ± 0.893	19.37 ± 0.526	2.01 ± 0.05	52.76 ± 4.616	6.44 ± 0.346	56.38 ± 1.429	16.03 ± 0.728
88.0	96.1 ± 0.227	99.44 ± 0.684	68.62 ± 4.973	32.45 ± 0.896	19.6 ± 0.563	2.0 ± 0.057	53.7 ± 4.715	6.46 ± 0.36	57.11 ± 1.629	16.11 ± 0.746
89.0	96.85 ± 0.087	99.43 ± 0.69	68.93 ± 4.984	32.57 ± 0.865	19.79 ± 0.617	2.02 ± 0.05	54.53 ± 4.804	6.49 ± 0.371	57.68 ± 1.829	16.18 ± 0.75
90.0	97.51 ± 0.095	99.47 ± 0.711	69.06 ± 4.909	32.65 ± 0.784	19.97 ± 0.66	2.01 ± 0.04	55.29 ± 4.937	6.51 ± 0.37	58.14 ± 1.998	16.22 ± 0.769
91.0	98.05 ± 0.098	99.44 ± 0.7	69.26 ± 4.969	32.75 ± 0.741	20.08 ± 0.682	2.01 ± 0.04	55.92 ± 4.995	6.53 ± 0.351	58.56 ± 2.148	16.28 ± 0.782
92.0	98.53 ± 0.026	99.43 ± 0.672	69.31 ± 5.025	32.82 ± 0.788	20.23 ± 0.721	2.01 ± 0.045	56.44 ± 5.058	6.55 ± 0.366	58.85 ± 2.281	16.3 ± 0.751
93.0	98.93 ± 0.042	99.37 ± 0.684	69.43 ± 5.031	32.87 ± 0.779	20.3 ± 0.715	2.02 ± 0.035	56.93 ± 5.118	6.57 ± 0.376	59.11 ± 2.379	16.34 ± 0.78
94.0	99.3 ± 0.083	99.4 ± 0.708	69.44 ± 5.028	32.95 ± 0.807	20.37 ± 0.794	2.02 ± 0.035	57.36 ± 5.155	6.58 ± 0.365	59.32 ± 2.489	16.37 ± 0.781
95.0	99.5 ± 0.086	99.31 ± 0.666	69.38 ± 5.078	33.01 ± 0.782	20.48 ± 0.781	2.03 ± 0.046	57.75 ± 5.195	6.6 ± 0.381	59.52 ± 2.553	16.39 ± 0.787
96.0	99.72 ± 0.055	99.32 ± 0.709	69.35 ± 5.043	33.06 ± 0.766	20.54 ± 0.767	2.01 ± 0.044	58.07 ± 5.238	6.59 ± 0.381	59.64 ± 2.653	16.42 ± 0.783
97.0	99.89 ± 0.085	99.22 ± 0.813	69.34 ± 4.989	33.12 ± 0.79	20.59 ± 0.835	2.02 ± 0.025	58.36 ± 5.267	6.62 ± 0.396	59.73 ± 2.695	16.43 ± 0.774
98.0	100.0 ± 0.095	99.17 ± 0.774	69.36 ± 4.953	33.15 ± 0.794	20.67 ± 0.824	2.02 ± 0.03	58.6 ± 5.327	6.62 ± 0.391	59.82 ± 2.757	16.44 ± 0.775
99.0	100.08 ± 0.118	99.12 ± 0.946	69.3 ± 4.945	33.19 ± 0.784	20.71 ± 0.836	2.03 ± 0.031	58.82 ± 5.336	6.63 ± 0.391	59.87 ± 2.8	16.46 ± 0.77
100.0	99.99 ± 0.132	99.14 ± 0.948	69.2 ± 4.879	33.21 ± 0.8	20.73 ± 0.848	2.02 ± 0.036	59.02 ± 5.408	6.63 ± 0.375	59.89 ± 2.916	16.48 ± 0.773
101.0	99.91 ± 0.134	99.26 ± 0.728	69.13 ± 4.803	33.26 ± 0.798	20.78 ± 0.805	2.02 ± 0.023	59.12 ± 5.387	6.64 ± 0.385	59.96 ± 2.871	16.49 ± 0.779
102.0	99.96 ± 0.095	99.2 ± 0.842	69.01 ± 4.683	33.28 ± 0.789	20.79 ± 0.855	2.03 ± 0.02	59.31 ± 5.415	6.65 ± 0.38	60.0 ± 2.885	16.49 ± 0.775
103.0	99.86 ± 0.137	99.21 ± 0.852	68.83 ± 4.57	33.32 ± 0.74	20.8 ± 0.851	2.03 ± 0.021	59.41 ± 5.425	6.66 ± 0.395	59.99 ± 2.922	16.5 ± 0.771
104.0	99.67 ± 0.184	99.08 ± 0.952	68.67 ± 4.489	33.32 ± 0.754	20.8 ± 0.847	2.03 ± 0.017	59.54 ± 5.439	6.66 ± 0.39	59.95 ± 2.898	16.52 ± 0.789
105.0	99.47 ± 0.191	99.1 ± 0.985	68.56 ± 4.422	33.35 ± 0.76	20.85 ± 0.885	2.03 ± 0.025	59.6 ± 5.428	6.67 ± 0.395	59.97 ± 2.925	16.51 ± 0.789
106.0	99.24 ± 0.34	99.07 ± 0.586	68.34 ± 4.408	33.33 ± 0.855	20.87 ± 0.898	2.03 ± 0.015	59.67 ± 5.468	6.67 ± 0.39	59.95 ± 2.924	16.53 ± 0.785
107.0	98.97 ± 0.292	98.92 ± 0.656	68.19 ± 4.312	33.39 ± 0.725	20.89 ± 0.885	2.04 ± 0.021	59.76 ± 5.473	6.68 ± 0.39	59.93 ± 2.913	16.53 ± 0.798
108.0	98.65 ± 0.486	98.84 ± 0.555	67.98 ± 4.25	33.42 ± 0.699	20.94 ± 0.882	2.04 ± 0.006	59.85 ± 5.5	6.67 ± 0.4	59.89 ± 2.93	16.53 ± 0.783

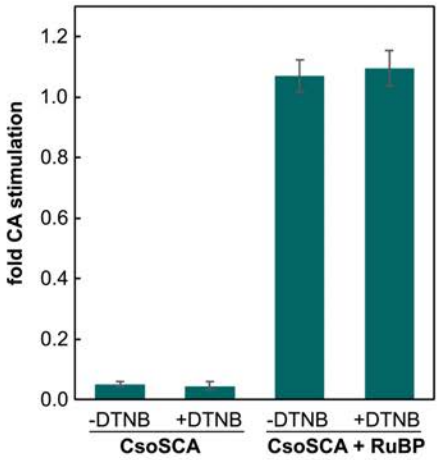

Figure S5 The oxidant DTNB does not affect RuBP activation of CsoSCA. CA activity was measured using the MIMS method in clarified lysate preparations of *E. coli* overexpressing CsoSCA extract before (-DTNB) and after incubation with 30µM DTNB (+DTNB) before (CsoSCA) and after addition of 100 µM RuBP (CsoSCA + RuBP). CA activity is reported as a proportion of the maximum recorded CA stimulation rate[7]. Values are means ± standard error of four technical replicates. RuBP dependent CA activity showed no statically significant differences between DTNB-treated and untreated CsoSCA extracts, as determined by a one-way ANOVA followed by Turkey's Honestly Significant Difference (HSD) test, p value between 'CsoSCA' samples with and without DTNB was calculated as 0.999 and for 'CsoSCA + RuBP' samples 0.981.

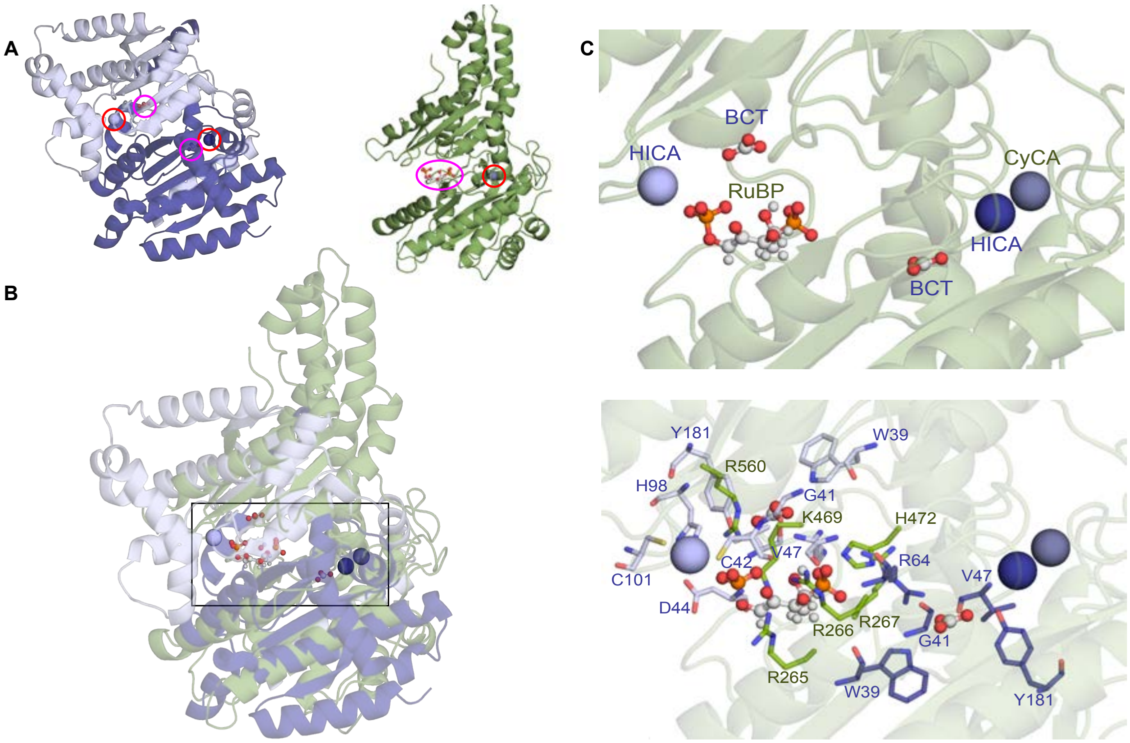

Figure S6. The RuBP binding site is distinct from previously identified allosteric bicarbonate site documented in Type II β-CAs, typified by the Haemophilus influenzae (HICA; PDB:2A8D)[36,77]. A The HICA dimer shown with monomers in different hues of purple and the *Cy*CsoSCA monomer (chain D) in green. The allosteric ligands are indicated by a magenta circle and the active zinc residues by a red circle in each structure. Type II β-CAs exhibit a pH-dependent activity profile symptomatic of an allosteric bicarbonate inhibition mechanism. Broadly, bicarbonate binding causes a conformational shift that disrupts the conserved Asp-Arg active site dyad, causing the Asp to coordinate the zinc ion and displace the catalytically essential water molecule[36,45,77]. The allosteric RuBP (*Cy*CsoSCA) and bicarbonate ions (HICA) are shown in ball and stick representation. Zinc ions are shown as spheres. **B** A structural alignment of the HICA dimer and the *Cy*CsoSCA monomer is shown with a box highlighting the zinc active sites and ligand binding pockets. **C** A box of the overlayed zinc ions and allosteric ligands. The RuBP ligand associated with *Cy*CsoSCA and bicarbonate ions (BCT) associated with HICA are shown in ball and stick representation and annotated. In the box below, ligand binding residues are shown in stick representation, purple residues correspond to sites in the HICA structure and green residues those in the *Cy*CsoSCA structure. The catalytic zinc binding residues C42, D44, H98 and C101 are shown the HICA monomer closest to the *Cy*CsoSCA RuBP binding site. Though in a similar region of the protein, the two sites employ different residues and the RuBP sits much further from the active

site relative to the allosteric bicarbonate site. These differences are largely due to the lack of active site pairing in the CsoSCA pseudo-dimer. While the fundamental unit of β-CAs is typically a dimer with symmetric active site pairing (or a pseudo-dimer in which the two monomers have fused but the active sites are each maintained), the CsoSCA clade has lost this defining feature[27]. Here, the C-terminal domain (CTD) does contain key structural motifs reminiscent of the Catalytic domain but has diverged so significantly that the active site is no longer intact.

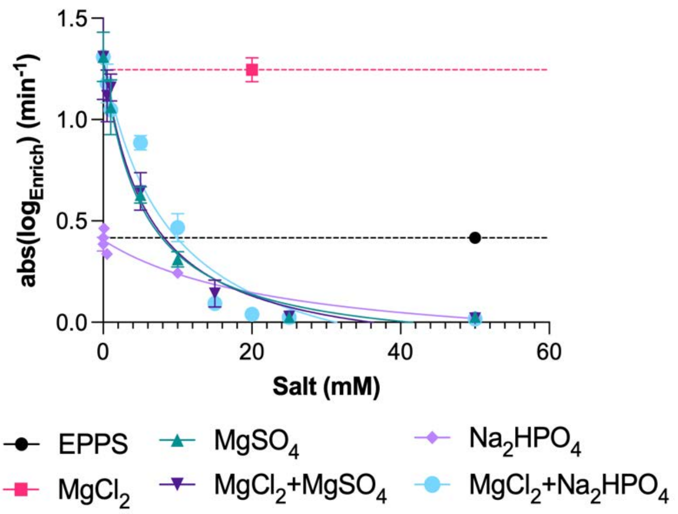

Figure S7. Given the overlaying positions of RuBP and sulfate ions, we hypothesised sulfate may compete with RuBP at this site, explaining low ligand density despite high concentrations in crystallisation conditions. *Cy*CsoSCA activity in the presence of sulfate and phosphate ions was quantified using MIMS[7] as detailed in the main text. All assays are run in the presence of 20mM EPPS. Activity in the absence of any salt (20mM EPPS) is shown, highlighting the requirement for Mg^{2+} to achieve maximal activity (50mM $MgCl_2$). Activity levels at this basal condition are indicated as single points/dashed horizontal lines. CA activity detected at increasing concentrations of key salts $MgSO_4$ and Na_2HPO_4 as shown in the figure legend are plotted. These results show *Cy*CsoSCA is significantly inhibited at 50mM $MgSO_4$ relative to standard assay conditions (20mM $MgCl_2$). Phosphate ions, a more biologically relevant ion with analogous size and charge, had a comparable effect to sulfate. *Cy*CsoSCA activity also dropped in the absence of Magnesium salts, perhaps functioning to stabilise the ligand in solution. It is unclear whether phosphate would ever reach millimolar concentrations within the carboxysome *in vivo*. All data points are the mean of three replicates, error bars denote standard error of the mean.

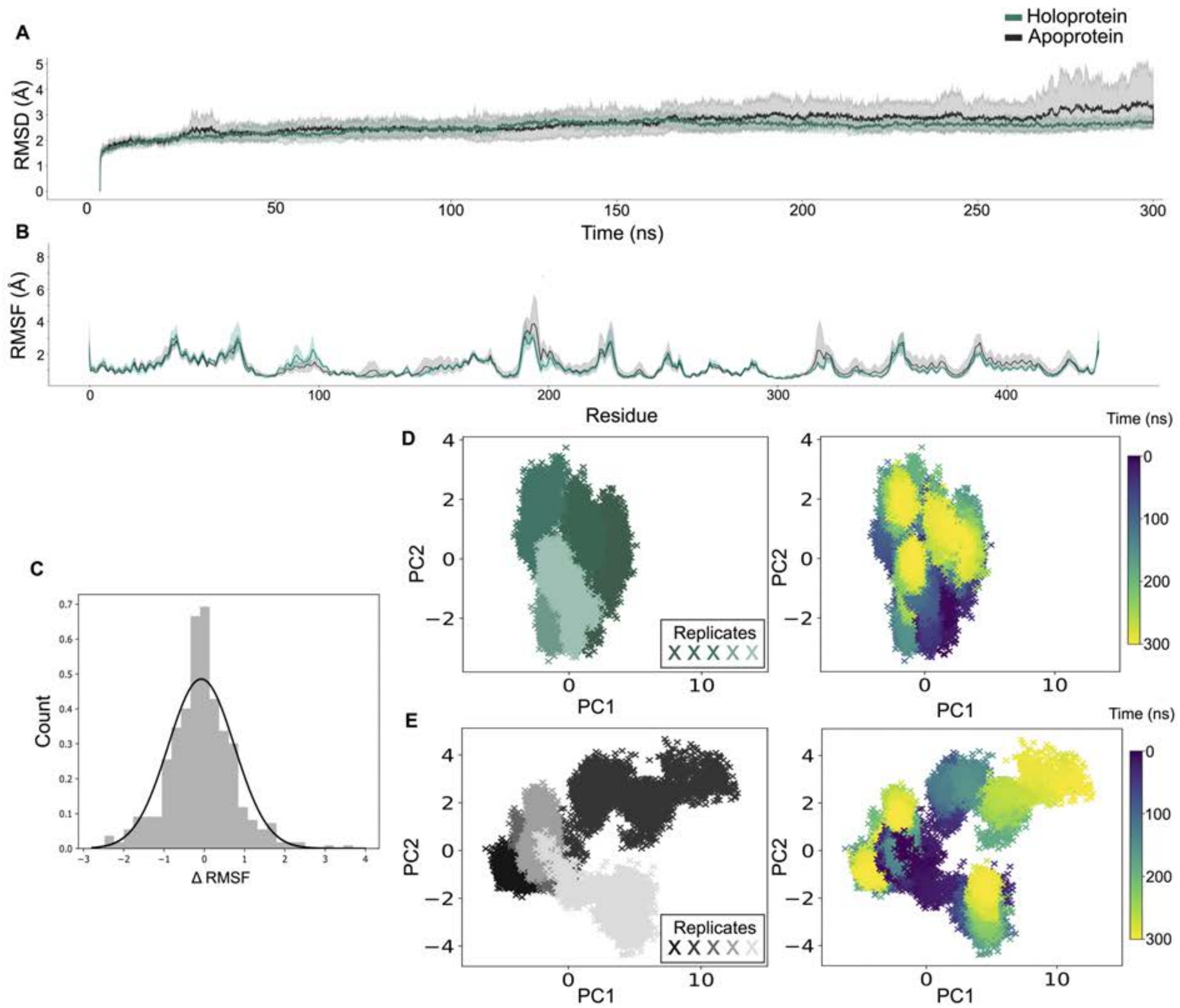

Figure S8. **Trajectory analysis of molecular dynamic simulations of CyCsoSCA Chain D with (holoenzyme) and without (apoenzyme) RuBP present. A** Cα RMSD (Å) variation across trajectory for each 300ns replicate relative to frame 1. Five replicates were conducted for each condition with frame recording intervals of 15ns, error indicates standard deviation. Both trajectories equilibrated, with a few fluctuations evident in the apoprotein trajectories particularly after 250ns. **B** Cα RMSF (Å) of each residue across the trajectory highlighting marginal site-by-site differences. **C** Histogram recording the difference between RMSF of analogous residues. Average RMSF (Å) was calculated for each residue and values from the apoenzyme trajectory were subtracted from the average RMSF value of the corresponding residue in the holoenzyme trajectory. Thus, a negative value is indicative of a residue with a greater RMSF in the apoenzyme trajectory and vice versa. These values have then been plotted onto the structure. Principal component analysis of cartesian coordinates of either holoprotein replicates (**D)** or apoprotein replicates (**E**) coloured by replicate (left) or by time (right). These PCA plots show that relative to apoprotein trajectories, the holoprotein occupies a much

narrower conformational space, indicative of more constrained conformational sampling. This corresponds with previous publications that conclude ligand binding stabilises the holoprotein, resulting a less flexibility relative to the apoprotein[79,80].

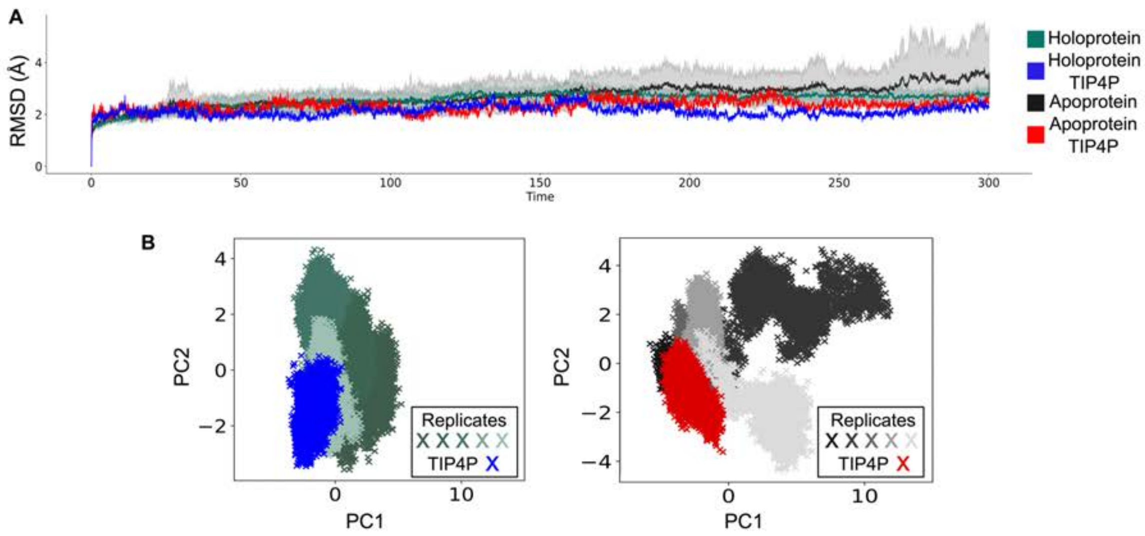

Figure S9 Additional molecular dynamics replicates with an alternate water model (TIP4P) compared to the existing five replicates. A Cα RMSD (Å) variation across trajectory for each 300ns replicate relative to frame 1 as presented in Fig S8A (holoprotein in green, apoprotein in black), the mean is plotted with standard error indicated by shading. Single trajectories with alternate water models of the apoprotein (red) and holoprotein (blue) are overlayed. **B** Principal component analyses (PCAs) of the holoprotein (left panel) and apoprotein (right panel) replicates with the relevant alternate water molecule simulations overlayed.

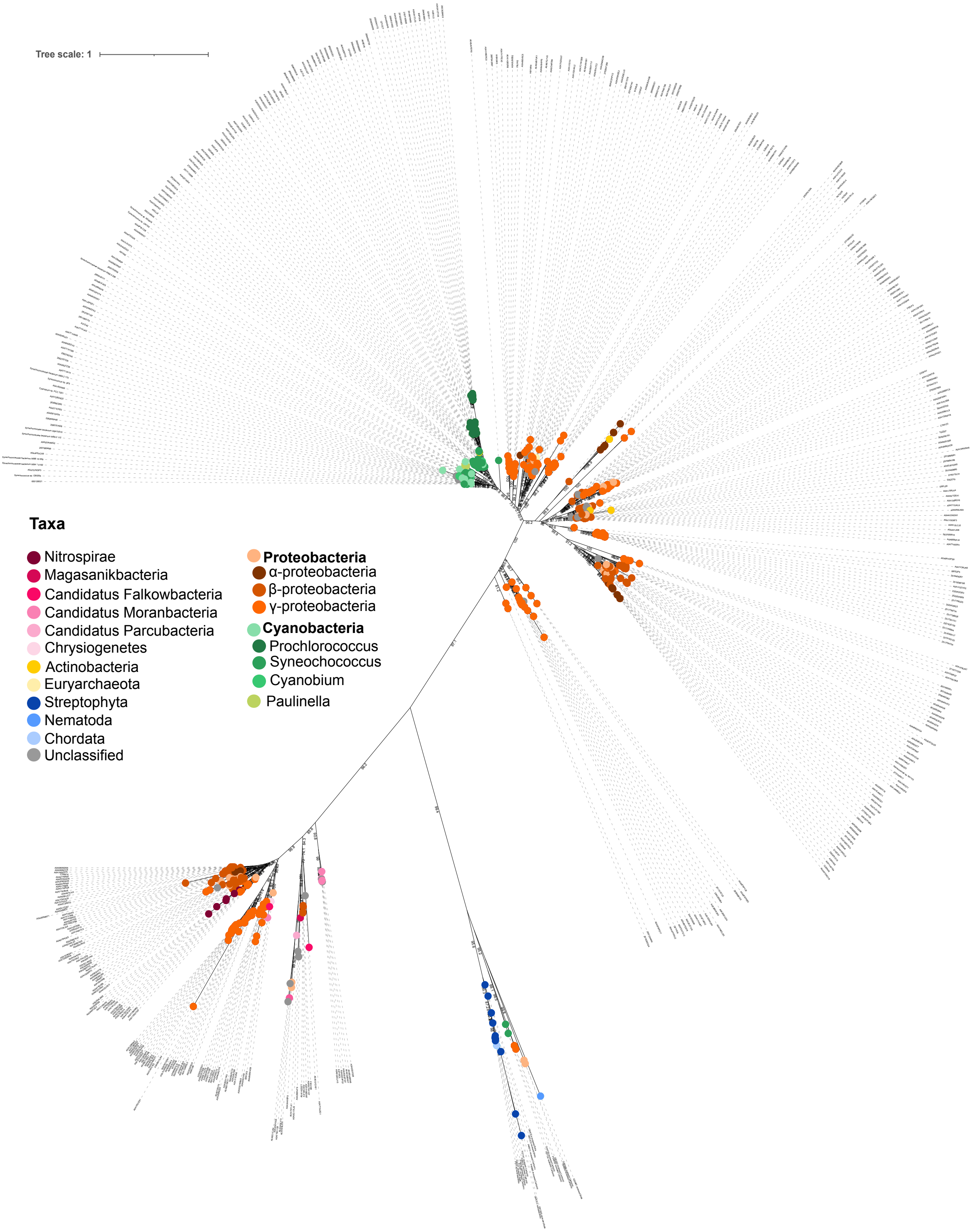

Figure S10. An unrooted phylogeny of the CsoSCA protein family inferred by maximum likelihood generated through IQ-Tree using standard parameters. Bootstrap values >70 generated using the ultrafast bootstrap approximation form 1000 replicates[8]. Extant nodes

coloured according to taxa as per key in the legend. A complete treefile, multiple sequence alignment, and final sequence annotations are provided as supplementary datafiles.

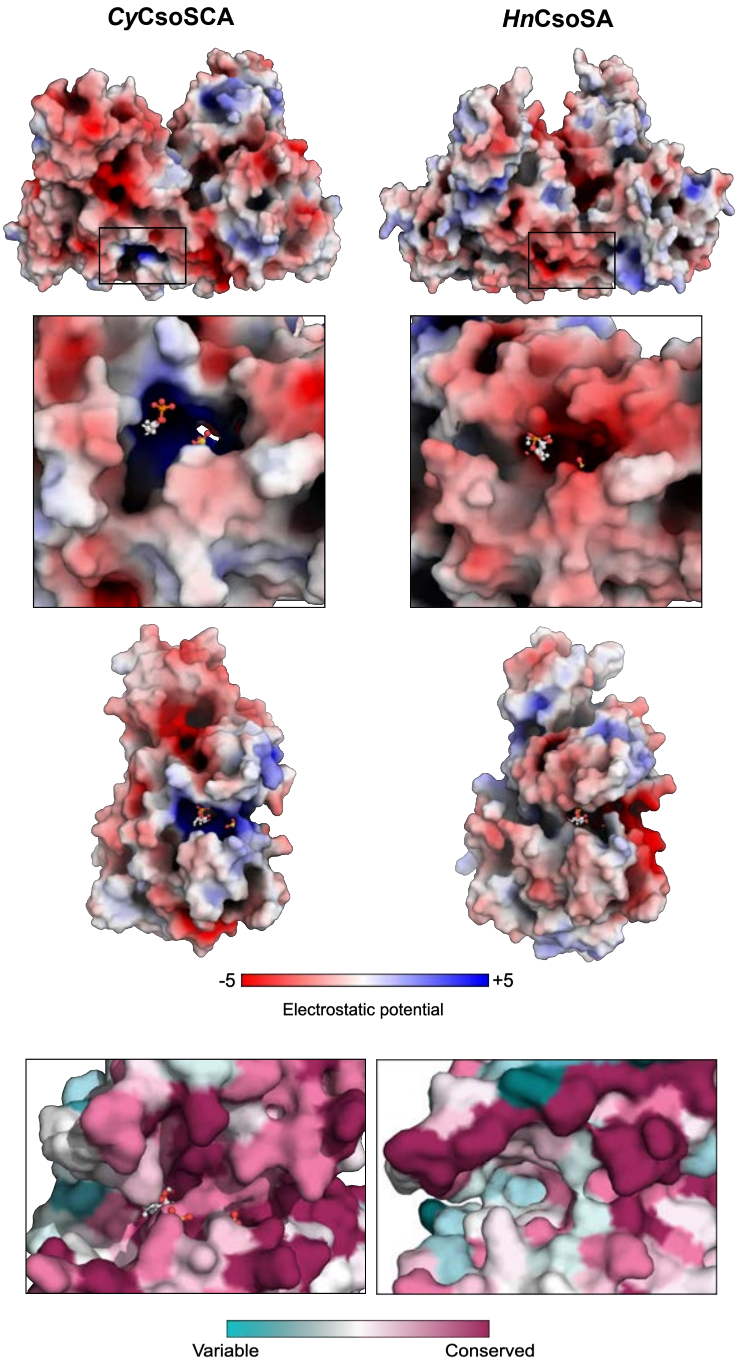

Figure S11. The RuBP pocket is conserved and positively charge in cyanobacterial CsoSCA variants. *Cy*CsoSCA and *Hn*CsoSCA structures coloured by electrostatic charge and residue conservation as per legends in the figure. From top to bottom, the dimer of each variant is shown, followed by a zoomed in box of the RuBP binding site, and then a view of the ligand pocket in the monomer. Given RuBP primarily exists in a negatively charged state[35], binding site charge was of key interest. In *Cy*CsoSCA, this site is positively charged, while the comparative region in the constitutively active *Hn*CsoSCA has a negative electrostatic

potential. The final panel displays a zoomed in box of the ligand pocket in each variant coloured by conservation. RuBP has been aligned onto the *Hn*CsoSCA for perspective. The multiple sequence alignment (MSA) of the curated CsoSCA dataset, comprising 134 cyanobacterial species and 337 other taxa, was used to assess the conservation of key features associated with RuBP binding across the protein family. Separate alignments of each taxonomic group were constructed and submitted to the ConSurf webserver for image generation[78]. The resulting conservation values were plotted onto the *Cy*CsoSCA structure and the *Hn*CsoSCA structure for cyanobacterial and other taxa alignments respectively. The positive charge lining the RuBP pocket in *Cy*CsoSCA appears to be a highly conserved feature among analysed cyanobacterial homologues. Comparatively, the conservation of the equivalent region in other taxa to the *Hn*CsoSCA structure, demonstrates the strong negative charge is not a conserved feature and that this region is quite variable across other taxa surveyed. This data is consistent with the positive charge of the region, and likely the capacity for RuBP binding, being conserved across α-cyanobacterial CsoSCAs but absent or un-conserved in other taxa.

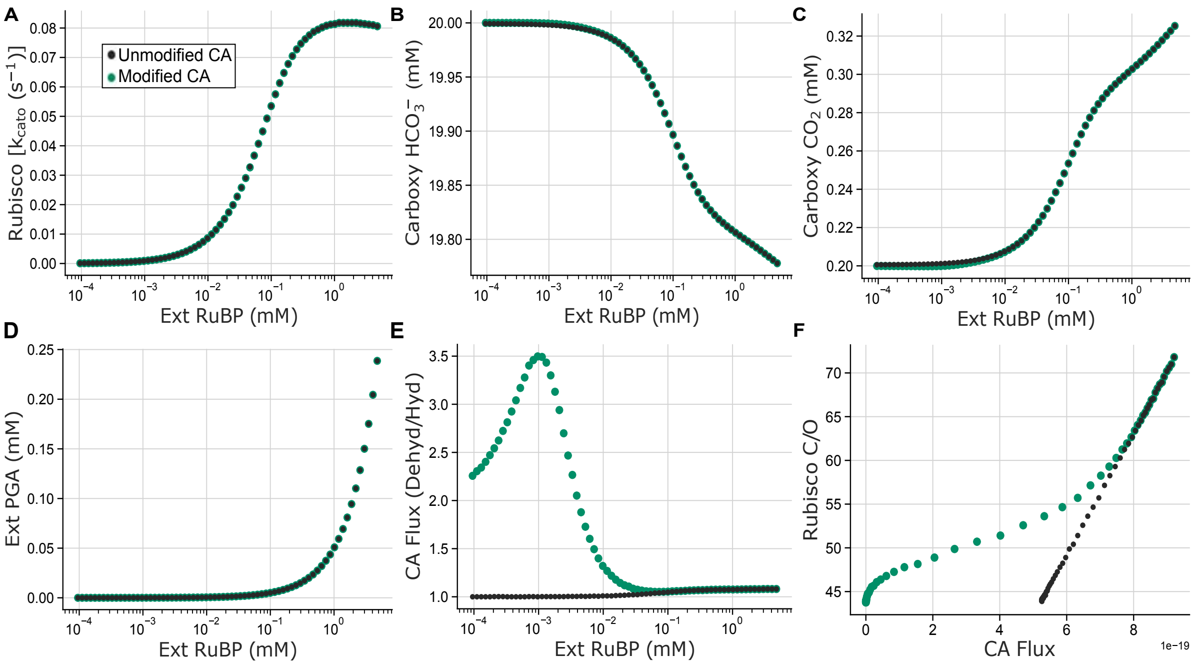

Figure S12. A model adapted from previous work simulating carboxysome function was adapted to emulate a *Cyanobium* α-carboxysome with either an RuBP-dependent CA (Modified CA, green dots) or a constitutively active CA (Unmodified CA, black dots)[35]. A Rubisco oxygenation activity per second (Rubisco (kcato) is equivalent between the two systems across modelled cellular RuBP concentrations (Ext RuBP (mM)), corresponding to the equivalent observed carboxylation rates (Figure 5). **B** The concentration of HCO_3^- and **C** CO_2 within the carboxysome as a function of modelled cellular RuBP concentrations (Ext RuBP (mM)). **D** Correspondingly, the concentration of modelled cellular PGA (Ext PGA (mM)) as a function of modelled cellular RuBP concentrations (Ext RuBP (mM)) demonstrates no difference in PGA production between the two systems. **E** While the carboxysomal CA is capable of both the forward and reverse CO_2 hydration/HCO_3^- dehydration reactions, to supply the carboxysome-encased Rubisco with CO_2 requires the dehydration reaction to predominate. The ratio of the fluxes recorded for the CA dehydration to hydration reaction (CA flux (Dehyd/Hyd)) is plotted here across a modelled cellular RuBP gradient (Ext RuBP (mM)). This demonstrates notable differences in CA flux and at low RuBP conditions in the two systems. As Ci appears consistent between each system, this likely manifests as changes in the carboxysomal proton concentration at these RuBP levels. Indeed, this is consistent with the observed changes in pH presented in Figure 5C. **F** The ratio of Rubisco carboxylation rates to oxygenation rates (Rubisco C/O) as CA activity (CA flux, indicative of CA activity levels converting HCO_3^- to CO_2) increases with cellular RuBP concentrations in the modified and unmodified systems.

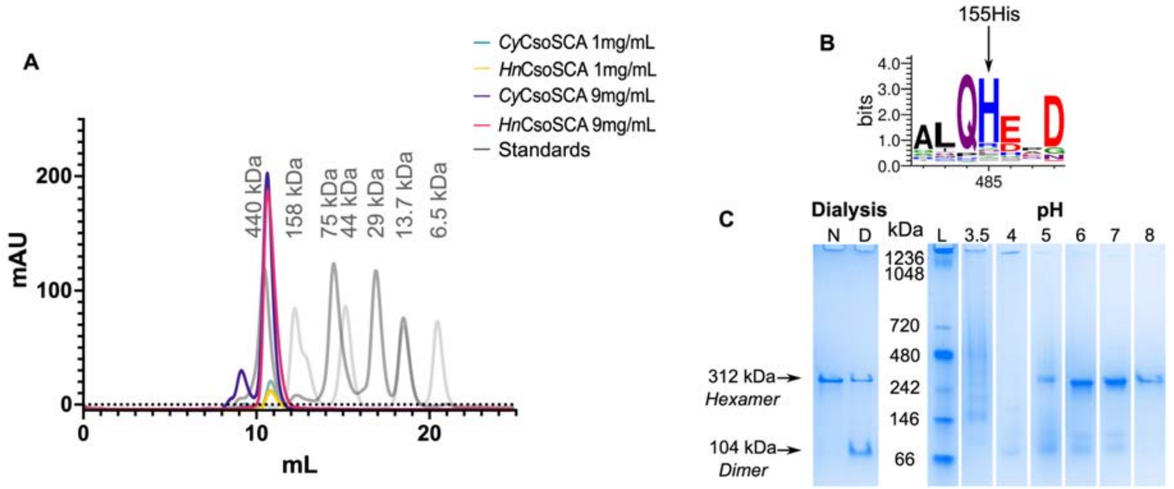

Figure S13. A homohexameric trimer of dimers is the prevailing, biologically relevant form of CsoSCA. A Size exclusion chromatography of *Cy*CsoSCA and *Hn*CsoSCA shows each isoform elutes at approximately the volume expected for the hexamer observed in the *Cy*CsoSCA crystal structure (approx. 312 kDa) independent of protein concentration. An additional peak in the *Cy*CsoSCA samples at both concentrations is also seen at slightly higher molecular weights suggesting, while the hexamer is the predominant form, a spectrum of oligomeric states may be present in solution. **B** A sequence logo with the key His155 residue required by all monomers to coordinate the structural zinc ion in an octahedral $His_3(H_2O)_3$ coordination sphere (Figure 1 main text). The key zinc coordination residue appears conserved across all analysed CsoSCA sequences. **C** To assess the role of the zinc ion in coordinating the hexamer, we perturbed zinc binding and observed the effect on oligomerisation. Native *Cy*CsoSCA (N) and *Cy*CsoSCA dialysed against 2mM 1,10-phenanthroline, a strong chelating agent, for 24 hours before being run on a Native PAGE to compare oligomeric states. This results in the breakdown of the high molecular weight band corresponding to the hexameric state, in favour of a smaller band approximately the size expected for the dimer. Additionally, *Cy*CsoSCA was incubated across a pH gradient and subsequently assessed by Native PAGE to capture pH dependent perturbations in oligomeric state. as solution pH approaches the pKa of Histidine a corresponding oligomeric shift is observed. At pH 3.5 smearing is indicative of protein denaturation and at pH 4 a faint band is visible between 146 kDa and 66 kDa consistent with the dimeric *Cy*CsoSCA (104 kDa). In samples incubated at pH 5, the dimeric band increases in intensity and a second band is evident between 242 kDa and 480 kDa consistent with the hexameric *Cy*CsoSCA (312 kDa). As the pH becomes more basic the hexameric band increases in intensity and the dimeric band fades. This would correspond with the hexamer destabilising as the Histidines coordinating the structural Zinc ions are protonated, and dimers

become the prevailing quaternary state in solution. At pH 6 and 7 a second faint band is evident above the dimeric band between the 66kDa and 146kDa markers. The distance between the two bands here is too small to denote a trimer thus it is likely this is some kind of contaminant. This data is consistent with the NTD acting as an oligomerisation domain, binding structural Zincs to facilitate the formation of large hexameric CsoSCA assemblies in solution. The standard is labelled (L) with corresponding weights annotated in kDa.

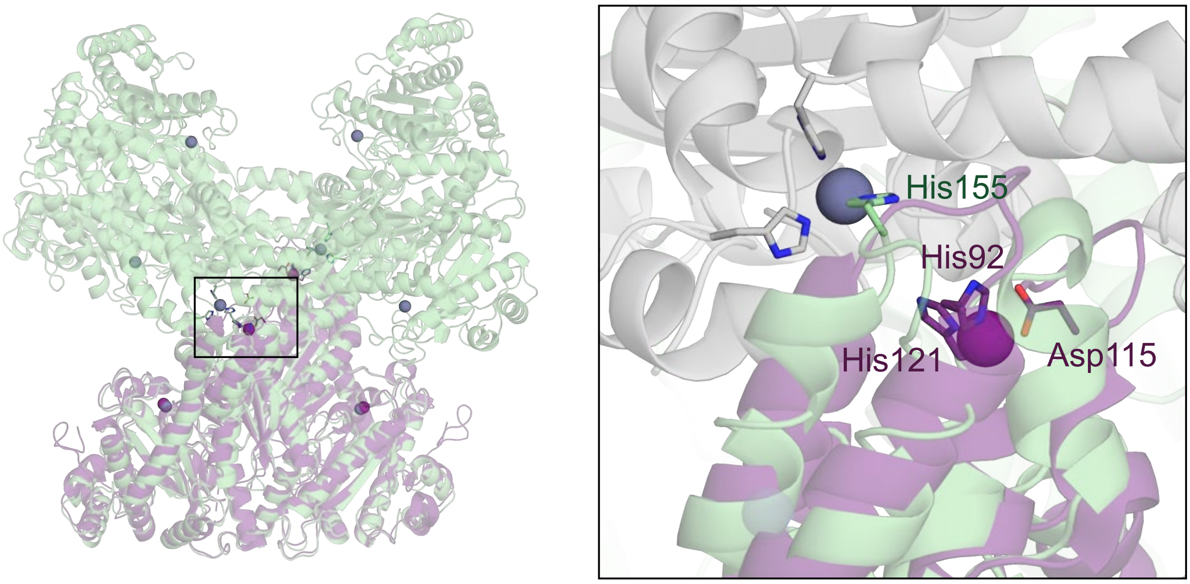

Figure S14. The opportune zinc site in the NTD of the crystallised *Hn*CsoSCA previously characterised arose from an inadvertent mutant and likely precluded the formation of the true hexameric state[27]. The HnCsoSCA dimer (PDB:2FGY) is shown (purple) overlaid with the *Cy*CsoSCA structure solved here (green) with zinc ligands shown as spheres (grey spheres for *Cy*CsoSCA associated Zincs and purple spheres for those associated with HnCsoSCA). A box with a zoomed in view of the structural zinc site is shown with key zinc binding ligands in *Cy*CsoSCA (His155) and *Hn*CsoSCA (His92, Asp115, His121) shown in stick representation. The purple sphere shown here depicts the non-biologically relevant zinc-binding site in the NTD while the grey sphere denotes the additional zinc ion demonstrated to mediate additional contacts necessary for hexamer formation.

Table S5. DALI search results for a non-*cso* sequence (UniProt ID A0A2N1ALC1).

Chain	Z score	RMSD	Lali	No.res	%ID	Description
**2fgy-a**	31.4	2.8	340	471	18	Carboxysome shell polypeptide
**1ddz-a**	17.5	3.4	273	481	14	Carbonic anhydrase;
**3vrk-a**	10.7	2.7	128	212	12	Carbonyl sulfide hydrolase;
**3vqj-a**	10.6	2.7	128	213	12	Carbonyl sulfide hydrolase;
**6gwu-b**	10.6	3.4	141	205	11	Carbonic anhydrase;
**2a5v-c**	10.4	2.7	131	201	6	Carbonic anhydrase (carbonate dehydratase)(carbo)

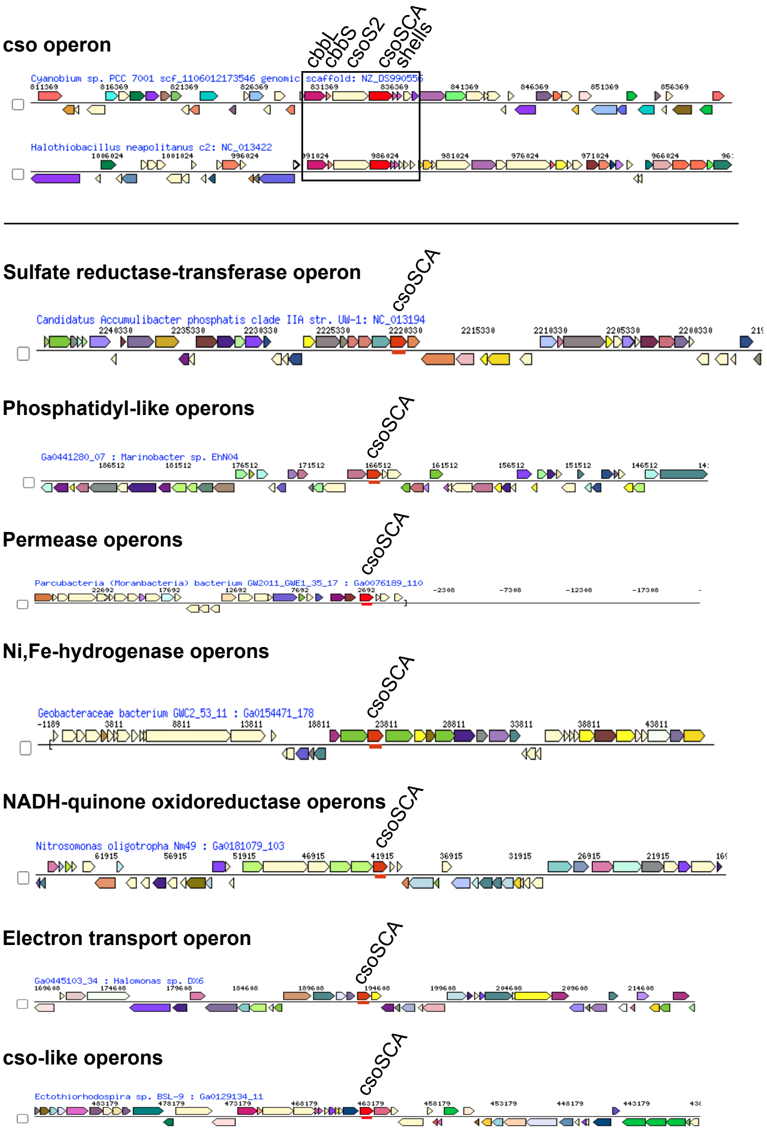

Figure S15. Example of operon types observed in the CsoSCA dataset. For each sequence in the 'non-cso' cluster the genomic context was pulled using the JGI IMG database and manually assessed. Canonical *Hn*CsoSCA and *Cy*CsoSCA demonstrate typical *cso* operon structure. The colours of each gene are automatically generated on JGI IMG based on different COG annotations.

Table S6. Primers used in this work to sequence and generate *Cy*CsoSCA mutants.

Target gene	Purpose	Destination Vector	Primer	Sequence
Cyanobium csoSCA	epPCR	pET16	csoS3_epPCR_F_BsaI	TAGGTCTCTCATATGCCGCGTCGTACC
			csoS3_epPCR_R_BsaI	TAGGTCTCTTCGATTAGTGACCGCCGCC
	Sequencing epPCR mutants		pET16_Seq_F	ATCTCTATATCTCTATTTGACGGCT
			pET16_Seq_R	CTAGGGCAAACATATAAACGCA
			CyCsoS3_sR1	TTACGCCAACATCCAGGTCTGC
	Creating H519Q CyCsoSCA mutant	pHue	Gibson_CA52_H519Q_F	CGTTTTGACTATCAGGGTCAGGTACCAGG
			Gibson_CA52_H519Q_R	CCTGGTACCTGACCCTGATAGTCAAAACG
	Creating T477A CyCsoSCA mutant		Gibson_CA52_T477A_F	GCGTAATCTGGCATACTTCGCTTACATGG
			Gibson_CA52_T477A_R	CCATGTAAGCGAAGTATGCCAGATTACGC
	Creating N278D CyCsoSCA mutant		Gibson_CA52_N278D_F	TGACGTTGAAAAAACCGTGAACCGTTGG
			Gibson_CA52_N278D_R	CCAACGGTTCACGGTTTTTTCAACGTCA

Legends for data S1 to S3

Data S1: Master csv of CsoSCA sequences used in analyses.
Data S2: Treefile for final inferred CsoSCA maximum likelihood phylogeny.
Data S3: Multiple sequence alignment used to infer phylogeny.

REFERENCES AND NOTES

1. J. A. Raven, C. S. Cockell, C. L. De La Rocha, The evolution of inorganic carbon concentrating mechanisms in photosynthesis. *Philos. Trans. R. Soc. Lond. B Biol. Sci.* **363**, 2641–2650 (2008).

2. M. R. Badger, G. D. Price, The CO_2 concentrating mechanism in cyanobactiria and microalgae. *Physiol. Plant.* **84**, 606–615 (1992).

3. M. R. Badger, D. Hanson, G. D. Price, Evolution and diversity of CO_2 concentrating mechanisms in cyanobacteria. *Funct. Plant Biol.* **29**, 161–173 (2002).

4. G. G. B. Tcherkez, G. D. Farquhar, T. J. Andrews, Despite slow catalysis and confused substrate specificity, all ribulose bisphosphate carboxylases may be nearly perfectly optimized. *Proc. Natl. Acad. Sci.* **103**, 7246–7251 (2006).

5. P. J. Cabello-Yeves, D. J. Scanlan, C. Callieri, A. Picazo, L. Schallenberg, P. Huber, J. J. Roda-Garcia, M. Bartosiewicz, O. I. Belykh, I. V. Tikhonova, A. Orcello-Requena, P. M. De Prado, A. D. Millard, A. Camacho, F. Rodriguez-Valera, R. J. Puxty, α-Cyanobacteria possessing form IA RuBisCO globally dominate aquatic habitats. *ISME J.* **16**, 2421–2432 (2022).

6. M. Badger, The roles of carbonic anhydrases in photosynthetic CO_2 concentrating mechanisms. *Photosynth. Res.* **77**, 83 (2003), 94.

7. M. Sommer, F. Cai, M. Melnicki, C. A. Kerfeld, β-Carboxysome bioinformatics: Identification and evolution of new bacterial microcompartment protein gene classes and core locus constraints. *J. Exp. Bot.* **68**, 3841–3855 (2017).

8. B. D. Rae, B. M. Long, M. R. Badger, G. D. Price, Functions, compositions, and evolution of the two types of carboxysomes: Polyhedral microcompartments that facilitate CO_2 fixation in cyanobacteria and some proteobacteria. *Microbiol. Mol. Biol. Rev.* **77**, 357–379 (2013).

9. M. R. Badger, G. D. Price, B. M. Long, F. J. Woodger, The environmental plasticity and ecological genomics of the cyanobacterial CO_2 concentrating mechanism. *J. Exp. Bot.* **57**, 249–265 (2006).

10. J. A. Raven, J. Beardall, CO_2 concentrating mechanisms and environmental change. *Aquat. Bot.* **118**, 24–37 (2014).

11. S. Fang, X. Huang, X. Zhang, M. Zhang, Y. Hao, H. Guo, L.-N. Liu, F. Yu, P. Zhang Molecular mechanism underlying transport and allosteric inhibition of bicarbonate transporter SbtA. *Proc. Natl. Acad. Sci. U.S.A.* **118**, e2101632118 (2021).

12. J. A. Kaczmarski, N.-S. Hong, B. Mukherjee, L. T. Wey, L. Rourke, B. Förster, T. S. Peat, G. D. Price, C. J. Jackson Structural basis for the allosteric regulation of the SbtA bicarbonate transporter by the PII-like protein, SbtB, from *Cyanobium* sp. PCC7001. *Biochemistry* **58**, 5030–5039 (2019).

13. F. J. Woodger, M. R. Badger, G. D. Price, Inorganic carbon limitation induces transcripts encoding components of the CO_2-concentrating mechanism in Synechococcus sp. PCC7942 through a redox-independent pathway. *Plant Physiol.* **133**, 2069–2080 (2003).

14. L. Whitehead, B. M. Long, G. D. Price, M. R. Badger, Comparing the in vivo function of α-carboxysomes and β-carboxysomes in two model cyanobacteria. *Plant Physiol.* **165**, 398–411 (2014).

15. Y. Sun, S. Casella, Y. Fang, F. Huang, M. Faulkner, S. Barrett, L.-N. Liu Light modulates the biosynthesis and organization of cyanobacterial carbon fixation machinery through photosynthetic electron flow. *Plant Physiol.* **171**, 530–541 (2016).

16. A. Rohnke Brandon, S. P. Singh, B. Pattanaik, B. L. Montgomery, RcaE-dependent regulation of carboxysome structural proteins has a central role in environmental determination of carboxysome morphology and abundance in Fremyella diplosiphon. *mSphere* **3**, e00617–17 (2018).

17. Y. Sun, A. J. M. Wollman, F. Huang, M. C. Leake, L.-N. Liu Single-organelle quantification reveals stoichiometric and structural variability of carboxysomes dependent on the environment. *Plant Cell* **31**, 1648–1664 (2019).

18. K. Harano, H. Ishida, R. Kittaka, K. Kojima, N. Inoue, M. Tsukamoto, R. Satoh, M. Himeno, T. Iwaki, A. Wadano Regulation of the expression of ribulose-1,5-bisphosphate carboxylase/oxygenase (EC 4.1.1.39) in a cyanobacterium, Synechococcus PCC7942. *Photosynth. Res.* **78**, 59–65 (2003).

19. F. Huang, O. Vasieva, Y. Sun, M. Faulkner, G. F. Dykes, Z. Zhao, L.-N. Liu Roles of RbcX in carboxysome biosynthesis in the cyanobacterium *Synechococcus elongatus* PCC7942. *Plant Physiol.* **179**, 184–194 (2019).

20. Y.-C. C. Tsai, M. C. Lapina, S. Bhushan, O. Mueller-Cajar Identification and characterization of multiple rubisco activases in chemoautotrophic bacteria. *Nat. Commun.* **6**, 8883 (2015).

21. M. Sutter, E. W. Roberts, R. C. Gonzalez, C. Bates, S. Dawoud, K. Landry, G. C. Cannon, S. Heinhorst, C. A. Kerfeld Structural characterization of a newly identified component of α-carboxysomes: The AAA+ domain protein CsoCbbQ. *Sci. Rep.* **5**, 16243 (2015).

22. K. L. Peña, S. E. Castel, C. de Araujo, G. S. Espie, M. S. Kimber Structural basis of the oxidative activation of the carboxysomal γ-carbonic anhydrase, CcmM, *Proc. Natl. Acad. Sci. U S A* **107**, 2455–2460 (2010).

23. K. S. Smith, C. Jakubzick, T. S. Whittam, J. G. Ferry Carbonic anhydrase is an ancient enzyme widespread in prokaryotes. *Proc. Natl. Acad. Sci.* **96**, 15184–15189 (1999).

24. A. U. Igamberdiev, Control of Rubisco function via homeostatic equilibration of CO_2 supply. *Front. Plant Sci.* **6**, (2015).

25. G. D. Price, M. R. Badger, Expression of human carbonic anhydrase in the cyanobacterium Synechococcus PCC7942 creates a high CO2-requiring phenotype. *Plant Physiol.* **91**, 505–513 (1989).

26. M. R. Badger, G. D. Price, G. D. Carbonic anhydrase activity associated with the cyanobacterium Synechococcus PCC7942. *Plant Physiol.* **89**, 51–60 (1989).

27. M. R. Sawaya, G. C. Cannon, S. Heinhorst, S. Tanaka, E. B. Williams, T. O. Yeates, C. A. Kerfeld The structure of β-carbonic anhydrase from the carboxysomal shell reveals a distinct subclass with one active site for the price of two. *J. Biol. Chem.* **281**, 7546–7555 (2006).

28. S. Heinhorst, E. B. Williams, F. Cai, C. D. Murin, J. M. Shively, G. C. Cannon Characterization of the carboxysomal carbonic anhydrase CsoSCA from *Halothiobacillus neapolitanus*. *J. Bacteriol.* **188**, 8087–8094 (2006).

29. T. Ni, Y. Sun, W. Burn, M. M. J. al-Hazeem, Y. Zhu, X. Yu, L.-N. Liu, P. Zhang Structure and assembly of cargo Rubisco in two native α-carboxysomes. *Nat. Commun.* **13**, 4299 (2022).

30. C. Blikstad, E. J. Dugan, T. G. Laughlin, M. D. Liu, S. R. Shoemaker, J. P. Remis, D. F. Savage, Discovery of a carbonic anhydrase-Rubisco supercomplex within the alpha-carboxysome. bioRxiv 467472 [Preprint] (2021). https://doi.org/10.1101/2021.11.05.467472.

31. L. A. Metskas, D. Ortega, L. M. Oltrogge, C. Blikstad, D. R. Lovejoy, T. G. Laughlin, D. F. Savage, G. J. Jensen Rubisco forms a lattice inside alpha-carboxysomes. *Nat. Commun.* **13**, 4863 (2022).

32. S. L. Evans, M. M. J. Al-Hazeem, D. Mann, N. Smetacek, A. J. Beavil, Y. Sun, T. Chen, G. F. Dykes, L.-N. Liu, J. R. C. Bergeron, Single-particle cryo-EM analysis of the shell architecture and internal organization of an intact α-carboxysome. bioRxiv 481072 [Preprint] (2022). https://doi.org/10.1101/2022.02.18.481072.

33. Y. Sun, V. M. Harman, J. R. Johnson, P. J. Brownridge, T. Chen, G. F. Dykes, Y. Lin, R. J. Beynon, L.-N. Liu Decoding the absolute stoichiometric composition and structural plasticity of α-carboxysomes. *MBio* **13**, e0362921 (2022).

34. T. Chaijarasphong, R. J. Nichols, K. E. Kortright, C. F. Nixon, P. K. Teng, L. M. Oltrogge, D. F. Savage Programmed ribosomal frameshifting mediates expression of the α-carboxysome. *J. Mol. Biol.* **428**, 153–164 (2016).

35. B. M. Long, B. Förster, S. B. Pulsford, G. D. Price, M. R. Badger Rubisco proton production can drive the elevation of CO_2 within condensates and carboxysomes. *Proc. Natl. Acad. Sci. U S A* **118**, e2014406118 (2021).

36. J. D. Cronk, R. S. Rowlett, K. Y. J. Zhang, C. Tu, J. A. Endrizzi, J. Lee, P. C. Gareiss, J. R. Preiss Identification of a novel noncatalytic bicarbonate binding site in eubacterial β-carbonic anhydrase. *Biochemistry* **45**, 4351–4361 (2006).

37. B. Marin, E. C. M. Nowack, G. Glöckner, M. Melkonian The ancestor of the Paulinella chromatophore obtained a carboxysomal operon by horizontal gene transfer from a Nitrococcus-like γ-proteobacterium. *BMC Evol. Biol.* **7**, 85 (2007).

38. B. Rae, B. Forster, M. Badger, G. Price, The CO_2-concentrating mechanism of Synechococcus WH5701 is composed of native and horizontally-acquired components. *Photosynth. Res.* **109**, 59–72 (2011).

39. B. Förster, L. M. Rourke, H. N. Weerasooriya, I. C. M. Pabuayon, V. Rolland, E. K. Au, S. Bala, J. Bajsa-Hirschel, S. Kaines, R. W. Kasili, L. M. LaPlace, M. C. Machingura, B. Massey, V. C. Rosati, H. Stuart-Williams, M. R. Badger, G. D. Price, J. V. Moroney The *Chlamydomonas reinhardtii* chloroplast envelope protein LCIA transports bicarbonate in planta. *J. Exp. Bot.* **74**, 3651–3666 (2023).

40. M. van Kempen, S. S. Kim, C. Tumescheit, M. Mirdita, J. Lee, C. L. M. Gilchrist, J. Söding, M. Steinegger, Fast and accurate protein structure search with Foldseek. bioRxiv 479398 [Preprint] (2023). https://doi.org/10.1101/2022.02.07.479398.

41. L. Holm, Using Dali for protein structure comparison, in *Structural Bioinformatics: Methods and Protocols*, Z. Gáspári, Ed. (Springer US, 2020), pp. 29–42.

42. M. R. Badger, Kinetic properties of ribulose 1,5-bisphosphate carboxylase/oxygenase from *Anabaena variabilis*. *Arch. Biochem. Biophys.* **201**, 247–254 (1980).

43. N. M. Mangan, A. Flamholz, R. D. Hood, R. Milo, D. F. Savage pH determines the energetic efficiency of the cyanobacterial CO_2 concentrating mechanism. *Proc. Natl. Acad. Sci. U.S.A.* **113**, E5354–E5362 (2016).

44. A. Flamholz, P. M. Shih, Cell biology of photosynthesis over geologic time. *Curr. Biol.* **30**, R490–R494 (2020).

45. R. S. Rowlett, C. Tu, J. Lee, A. G. Herman, D. A. Chapnick, S. H. Shah, P. C. Gareiss Allosteric site variants of Haemophilus influenzae β-carbonic anhydrase. *Biochemistry* **48**, 6146–6156 (2009).

46. S. Kim, J. Yeon, J. Sung, N. J. Kim, S. Hong, M. S. Jin Structural insights into novel mechanisms of inhibition of the major β-carbonic anhydrase CafB from the pathogenic fungus Aspergillus fumigatus. *J. Struct. Biol.* **213**, 107700 (2021).

47. A. S. Covarrubias, T. Bergfors, T. A. Jones, M. Högbom. Structural mechanics of the pH-dependent activity of β-carbonic anhydrase from *Mycobacterium tuberculosis*. *J. Biol. Chem.* **281**, 4993–4999 (2006).

48. A. E. Todd, C. A. Orengo, J. M. Thornton, Evolution of function in protein superfamilies, from a structural perspective. *J. Mol. Biol.* **307**, 1113–1143 (2001).

49. K. Fujiwara, H. Toda, M. Ikeguchi, Dependence of α-helical and β-sheet amino acid propensities on the overall protein fold type. *BMC Struct. Biol.* **12**, 18 (2012).

50. L. D. McGurn, M. Moazami-Goudarzi, S. A. White, T. Suwal, B. Brar, J. Q. Tang, G. S. Espie, M. S. Kimber The structure, kinetics and interactions of the β-carboxysomal β-carbonic anhydrase, CcaA, *Biochem. J.* **473**, 4559–4572 (2016).

51. O. Mueller-Cajar, M. Stotz, P. Wendler, F. U. Hartl, A. Bracher, M. Hayer-Hartl Structure and function of the AAA+ protein CbbX, a red-type Rubisco activase. *Nature* **479**, 194–199 (2011).

52. K. P. Dobrinski, D. L. Longo, K. M. Scott The carbon-concentrating mechanism of the hydrothermal vent chemolithoautotroph *Thiomicrospira crunogena*. *J. Bacteriol.* **187**, 5761–5766 (2005).

53. C. de Araujo, D. Arefeen, Y. Tadesse, B. M. Long, G. D. Price, R. S. Rowlett, M. S. Kimber, G. S. Espie Identification and characterization of a carboxysomal γ-carbonic anhydrase from the cyanobacterium Nostoc sp. PCC 7120. *Photosynth. Res.* **121**, 135–150 (2014).

54. L. M. Oltrogge, T. Chaijarasphong, A. W. Chen, E. R. Bolin, S. Marqusee, D. F. Savage Multivalent interactions between CsoS2 and Rubisco mediate α-carboxysome formation. *Nat. Struct. Mol. Biol.* **27**, 281–287 (2020).

55. A. I. Flamholz, E. Dugan, J. Panich, J. J. Desmarais, L. M. Oltrogge, W. W. Fischer, S. W. Singer, D. F. Savage Trajectories for the evolution of bacterial CO_2-concentrating mechanisms. *Proc. Natl. Acad. Sci.* **119**, e2210539119 (2022).

56. A.-M. Catanzariti, T. A. Soboleva, D. A. Jans, P. G. Board, R. T. Baker An efficient system for high-level expression and easy purification of authentic recombinant proteins. *Protein Sci.* **13**, 1331–1339 (2004).

57. C. Aussignargues, B. C. Paasch, R. Gonzalez-Esquer, O. Erbilgin, C. A. Kerfeld Bacterial microcompartment assembly: The key role of encapsulation peptides. *Commun. Integr. Biol.* **8**, e1039755 (2015).

58. D. G. Gibson, L. Young, R.-Y. Chuang, J. C. Venter, C. A. Hutchison III, H. O. Smith Enzymatic assembly of DNA molecules up to several hundred kilobases. *Nat. Methods* **6**, 343–345 (2009).

59. M. R. Wilkins, E. Gasteiger, A. Bairoch, J. C. Sanchez, K. L. Williams, R. D. Appel, D. F. Hochstrasser, Protein identification and analysis tools in the ExPASy server. *Methods Mol. Biol.* **112**, 531–552 (1999).

60. J. Jumper, R. Evans, A. Pritzel, T. Green, M. Figurnov, O. Ronneberger, K. Tunyasuvunakool, R. Bates, A. Žídek, A. Potapenko, A. Bridgland, C. Meyer, S. A. A. Kohl, A. J. Ballard, A. Cowie, B. Romera-Paredes, S. Nikolov, R. Jain, J. Adler, T. Back, S. Petersen, D. Reiman, E. Clancy, M. Zielinski, M. Steinegger, M. Pacholska, T. Berghammer, S. Bodenstein, D. Silver, O. Vinyals, A. W. Senior, K. Kavukcuoglu, P. Kohli, D. Hassabis Highly accurate protein structure prediction with AlphaFold. *Nature* **596**, 583–589 (2021).

61. M. Mirdita, K. Schütze, Y. Moriwaki, L. Heo, S. Ovchinnikov, M. Steinegger ColabFold: Making protein folding accessible to all. *Nat. Methods* **19**, 679–682 (2022).

62. P. Emsley, K. Cowtan, Coot: Model-building tools for molecular graphics. *Acta Crystallogr. D Biol. Crystallogr.* **60**, 2126–2132 (2004).

63. J. Agirre, M. Atanasova, H. Bagdonas, C. B. Ballard, A. Baslé, J. Beilsten-Edmands, R. J. Borges, D. G. Brown, J. J. Burgos-Mármol, J. M. Berrisford, P. S. Bond, I. Caballero, L. Catapano, G. Chojnowski, A. G. Cook, K. D. Cowtan, T. I. Croll, J. É. Debreczeni, N. E. Devenish, E. J. Dodson, T. R. Drevon, P. Emsley, G. Evans, P. R. Evans, M. Fando, J. Foadi, L. Fuentes-Montero, E. F. Garman, M. Gerstel, R. J. Gildea, K. Hatti, M. L. Hekkelman, P. Heuser, S. W. Hoh, M. A. Hough, H. T. Jenkins, E. Jiménez, R.

P. Joosten, R. M. Keegan, N. Keep, E. B. Krissinel, P. Kolenko, O. Kovalevskiy, V. S. Lamzin, D. M. Lawson, A. A. Lebedev, A. G. W. Leslie, B. Lohkamp, F. Long, M. Malý, A. J. McCoy, S. J. McNicholas, A. Medina, C. Millán, J. W. Murray, G. N. Murshudov, R. A. Nicholls, M. E. M. Noble, R. Oeffner, N. S. Pannu, J. M. Parkhurst, N. Pearce, J. Pereira, A. Perrakis, H. R. Powell, R. J. Read, D. J. Rigden, W. Rochira, M. Sammito, F. Sánchez Rodríguez, G. M. Sheldrick, K. L. Shelley, F. Simkovic, A. J. Simpkin, P. Skubak, E. Sobolev, R. A. Steiner, K. Stevenson, I. Tews, J. M. H. Thomas, A. Thorn, J. T. Valls, V. Uski, I. Usón, A. Vagin, S. Velankar, M. Vollmar, H. Walden, D. Waterman, K. S. Wilson, M. D. Winn, G. Winter, M. Wojdyr, K. Yamashita The CCP4 suite: Integrative software for macromolecular crystallography. *Acta Crystallogr. Sect. Struct. Biol.* **79**, 449–461 (2023).

64. D. Liebschner, P. V. Afonine, M. L. Baker, G. Bunkóczi, V. B. Chen, T. I. Croll, B. Hintze, L.-W. Hung, S. Jain, A. J. McCoy, N. W. Moriarty, R. D. Oeffner, B. K. Poon, M. G. Prisant, R. J. Read, J. S. Richardson, D. C. Richardson, M. D. Sammito, O. V. Sobolev, D. H. Stockwell, T. C. Terwilliger, A. G. Urzhumtsev, L. L. Videau, C. J. Williams, P. D. Adams Macromolecular structure determination using X-rays, neutrons and electrons: Recent developments in Phenix. *Acta Crystallogr. Sect. Struct. Biol.* **75**, 861–877 (2019).

65. R. T. McGibbon, K. A. Beauchamp, M. P. Harrigan, C. Klein, J. M. Swails, C. X. Hernández, C. R. Schwantes, L.-P. Wang, T. J. Lane, V. S. Pande MDTraj: A modern open library for the analysis of molecular dynamics trajectories. *Biophys. J.* **109**, 1528–1532 (2015).

66. C. Camacho, G. Coulouris, V. Avagyan, N. Ma, J. Papadopoulos, K. Bealer, T. L. Madden BLAST+: Architecture and applications. *BMC Bioinformatics* **10**, 421 (2009).

67. L. Fu, B. Niu, Z. Zhu, S. Wu, W. Li CD-HIT: Accelerated for clustering the next-generation sequencing data. *Bioinformatics* **28**, 3150–3152 (2012).

68. K. Katoh, D. M. Standley, MAFFT multiple sequence alignment software version 7: Improvements in performance and usability. *Mol. Biol. Evol.* **30**, 772–780 (2013).

69. S. Capella-Gutiérrez, J. M. Silla-Martínez, T. Gabaldón trimAl: A tool for automated alignment trimming in large-scale phylogenetic analyses. *Bioinformatics* **25**, 1972–1973 (2009).

70. B. Q. Minh, H. A. Schmidt, O. Chernomor, D. Schrempf, M. D. Woodhams, A. von Haeseler, R. Lanfear IQ-TREE 2: New models and efficient methods for phylogenetic inference in the genomic era. *Mol. Biol. Evol.* **37**, 1530–1534 (2020).

71. D. T. Hoang, O. Chernomor, A. von Haeseler, B. Q. Minh, L. S. Vinh UFBoot2: Improving the ultrafast bootstrap approximation. *Mol. Biol. Evol.* **35**, 518–522 (2018).

72. I. Letunic, P. Bork, Interactive Tree Of Life (iTOL) v5: An online tool for phylogenetic tree display and annotation. *Nucleic Acids Res.* **49**, W293–W296 (2021).

73. G. E. Crooks, G. Hon, J.-M. Chandonia, S. E. Brenner WebLogo: A sequence logo generator. *Genome Res.* **14**, 1188–1190 (2004).

74. V. M. Markowitz, F. Korzeniewski, K. Palaniappan, E. Szeto, G. Werner, A. Padki, X. Zhao, I. Dubchak, P. Hugenholtz, I. Anderson, A. Lykidis, K. Mavromatis, N. Ivanova, N. C. Kyrpides The integrated microbial genomes (IMG) system. *Nucleic Acids Res.* **34**, D344–D348 (2006).

75. D. Liebschner, P. V. Afonine, N. W. Moriarty, B. K. Poon, O. V. Sobolev, T. C. Terwilliger, P. D. Adams Polder maps: Improving OMIT maps by excluding bulk solvent. *Acta Crystallogr. Sect. Struct. Biol.* **73**, 148–157 (2017).

76. E. L. Angleton, H. E. Van Wart, Preparation and reconstitution with divalent metal ions of class I and class II *Clostridium histolyticum* apocollagenases. *Biochemistry* **27**, 7406–7412 (1988).

77. J. D. Cronk, J. A. Endrizzi, M. R. Cronk, J. W. O'neill, K. Y. J. Zhang Crystal structure of *E. coli* β-carbonic anhydrase, an enzyme with an unusual pH-dependent activity. *Protein Sci.* **10**, 911–922 (2001).

78. B. Yariv, E. Yariv, A. Kessel, G. Masrati, A. B. Chorin, E. Martz, I. Mayrose, T. Pupko, N. Ben-Tal Using evolutionary data to make sense of macromolecules with a “face-lifted” ConSurf. *Protein Sci.* **32**, e4582 (2023).

79. D. A. Landfried, D. A. Vuletich, M. P. Pond, J. T. J. Lecomte Structural and thermodynamic consequences of b heme binding for monomeric apoglobins and other apoproteins. *Gene* **398**, 12–28 (2007).

80. J. J. Clark, M. L. Benson, R. D. Smith, H. A. Carlson Inherent versus induced protein flexibility: Comparisons within and between apo and holo structures. *PLOS Comput. Biol.* **15**, e1006705 (2019).
